# Laminar shear stress alleviates monocyte adhesion and atherosclerosis development via miR-29b-3p/CX3CL1 axis regulation

**DOI:** 10.1242/jcs.259696

**Published:** 2022-07-22

**Authors:** Luya Pu, Qingyu Meng, Shuai Li, Yaru Wang, Banghao Sun, Bin Liu, Fan Li

**Affiliations:** 1 Department of Pathogenobiology, The Key Laboratory of Zoonosis, Chinese Ministry of Education, College of Basic Medicine, Jilin University, Changchun, 130021, China; 2 Department of Immunology, School of Basic Medical Sciences, Xinjiang Medical University, Urumqi, Xinjiang, 830000, China; 3 Cardiovascular Disease Center, The First Hospital of Jilin University, Changchun, 130021, China; 4 Engineering Research Center for Medical Biomaterials of Jilin Province, Jilin University, Changchun, 130021, China; 5 Key Laboratory for Health Biomedical Materials of Jilin Province, Jilin University, Changchun, 130021, China; 6 State Key Laboratory of Pathogenesis, Prevention and Treatment of High Incidence Diseases in Central Asia, Xinjiang, 830000, China; 7 The Key Laboratory for Bionics Engineering, Ministry of Education, Jilin University, Changchun, 130021, China

**Keywords:** Laminar shear stress, Atherosclerosis, CX3CL1, Monocyte adhesion, MiR-29b-3p

## Abstract

Laminar shear stress (Lss) is an important anti-atherosclerosis (anti-AS) factor, but its mechanism network is not clear. Therefore, this study aimed to identify how Lss acts against AS formation from a new perspective. In this study, we analyzed high-throughput sequencing data from static and Lss-treated human aortic and human umbilical vein endothelial cells (HAECs and HUVECs, respectively) and found that the expression of *CX3CL1*, which is a target gene closely related to AS development, was lower in the Lss group. Lss alleviated the inflammatory response in TNF-α (also known as TNF)-activated HAECs by regulating the miR-29b-3p/CX3CL1 axis, and this was achieved by blocking nuclear factor (NF)-κB signaling. In complementary *in vivo* experiments, a high-fat diet (HFD) induced inflammatory infiltration and plaque formation in the aorta, both of which were significantly reduced after injection of agomir-miRNA-29b-3p via the tail vein into HFD-fed *ApoE*^−/−^ mice. In conclusion, this study reveals that the Lss-sensitive miR-29b-3p/CX3CL1 axis is an important regulatory target that affects vascular endothelial inflammation and AS development. Our study provides new insights into the prevention and treatment of AS.

## Abstract (precis)

**Summary:** The laminar shear stress-sensitive miR-29b-3p/CX3CL1 axis significantly inhibits monocyte adhesion to activated human aortic endothelial cells, and alleviates local inflammation and plaque formation in *ApoE*^−/−^ mice fed a high-fat diet.

## INTRODUCTION

Fluid shear stress is the friction generated on the vascular endothelium by blood flow. In response to blood flow, vascular endothelial cells convert perceived changes in fluid shear stress into biological signals, thereby regulating gene expression and cell behavior (Baeyens et al., 2016). Studies have reported that fluid shear stress regulates various physiological processes, including arterial endothelial permeability (Ogunrinade et al., 2002), vascular tension and pressure (Iring et al., 2019), vascular remodeling (Min and Schwartz, 2019) and endothelial progenitor cell differentiation (Obi et al., 2012). Therefore, fluid shear stress is a key factor affecting vascular physiology. Depending on the shape and location of blood flow, fluid shear stress has different manifestations. For example, when blood flows in a straight part of a blood vessel, laminar shear stress (Lss) is generated, but when blood flows through a vascular branch, bifurcation or bend, oscillating shear stress or turbulence is generated.

A large number of studies have confirmed that the incidence of atherosclerosis (AS) is low in straight blood vessels, whereas blood vessel bifurcations and bends demonstrate a high incidence of AS. Thus, it appears that Lss has an important anti-AS effect (Traub and Berk, 1998; Zhou et al., 2014; Pan, 2009). Yuan et al. have shown that Lss inhibits the Hippo/Yes-associated protein (YAP) pathway by inducing autophagy and SIRT1 expression, thereby alleviating endothelial cell inflammation and resisting AS plaque formation (Yuan et al., 2020). In addition, Lss interrupts AS progression by inhibiting endoplasmic reticulum stress-induced apoptosis of endothelial cells (Kim and Woo, 2018). Studies have also suggested that Lss exerts its anti-AS effect by directly inhibiting the expression of macrophage migration inhibitory factor (Qiao et al., 2018). After clarifying the anti-AS effect of Lss, this study further explored the mechanism of Lss against the occurrence and development of AS.

CX3CL1 is a macromolecular protein composed of 373 amino acids and is currently the only known member of the CX3C chemokine family (Luo et al., 2019). Unlike other chemokines, CX3CL1 exists in two forms (secreted and membrane-bound); thus, it has a dual function as a chemokine and an adhesion molecule (Liu et al., 2016). CX3CL1 is closely related to various systemic diseases, such as neurological diseases (Pawelec et al., 2020), bone metabolism diseases (Wojdasiewicz et al., 2019) and cardiovascular diseases (Apostolakis and Spandidos, 2013). Studies have proposed that CX3CL1 mediates monocyte migration and adhesion (Ancuta et al., 2003). Monocytes in the blood preferentially recruit to blood vessel walls with a high concentration of CX3CL1, which damages the function of endothelial cells and induces AS plaque formation (Lesnik et al., 2003). Studies have reported that low shear stress induces adhesion of monocytes to TNF-α (also known as TNF)-activated human umbilical vein endothelial cells (HUVECs) by activating CX3CL1, whereas physiological shear stress eliminates this endothelial inflammation (Babendreyer et al., 2017). By analyzing high-throughput sequencing data, we found that CX3CL1 was expressed at a low level in Lss-treated endothelial cells. Therefore, this study revealed that Lss inhibits AS progression by reducing CX3CL1 expression.

MicroRNAs (miRNAs) are a type of non-coding single-stranded RNA with a length of ∼22 nucleotides that participate in the regulation of gene expression after transcription (Lu and Rothenberg, 2018). An increasing amount of evidence indicates that miRNAs regulate various biological processes, such as cell growth, differentiation, development and apoptosis, thereby playing a key role in the occurrence and development of many diseases. Overexpression of miR-217 in *ApoE*^−/−^ mice significantly inhibits the activation of endothelial nitric oxide synthase and promotes endothelial cell dysfunction, thereby exacerbating AS development (de Yébenes et al., 2020). Vascular endothelial cells exposed to long-term Lss regulate the expression of chemokines and proteoglycans by upregulating miR-126, thereby affecting vascular remodeling (Mondadori dos Santos et al., 2015). Studies have proposed that Lss maintains stable endothelial cell function by regulating miRNAs, thereby exerting an anti-AS effect (Boon et al., 2012). In view of this, this study explored the effect of Lss-sensitive miR-29b-3p on endothelial cell function and AS formation.

This study combined bioinformatics, molecular biology, and other research methods to examine the Lss-sensitive miR-29b-3p/CX3CL1 axis. We explored the influence of this axis on endothelial cell inflammation and the occurrence and development of AS both *in vitro* and *in vivo*, thereby improving knowledge on the regulation of AS and providing new ideas and insights for the prevention and treatment of AS.

## RESULTS

### Downregulation of endothelial CX3CL1 expression in response to Lss

**Fig. 1.**
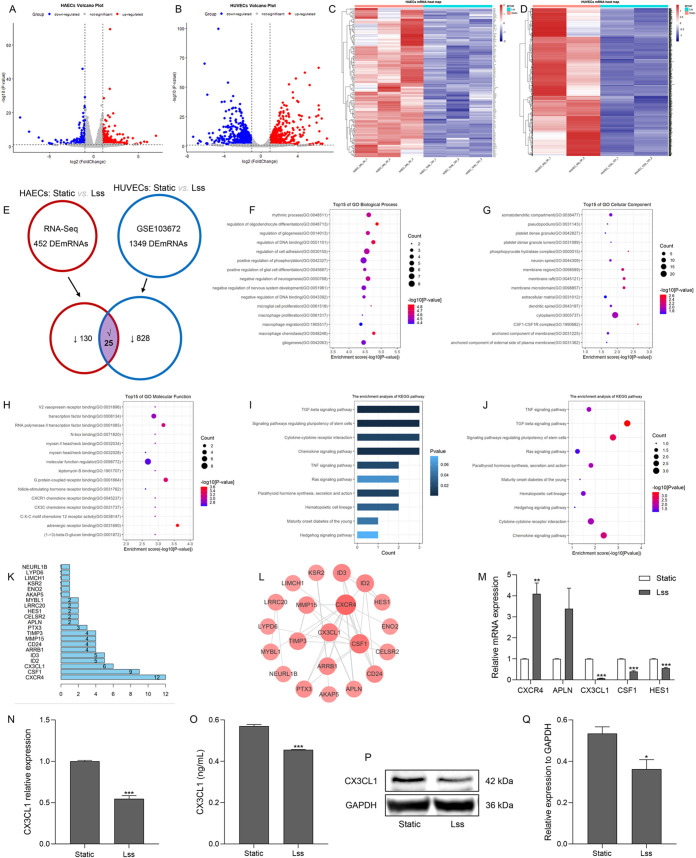
**Endothelial CX3CL1 expression is downregulated under Lss.** Volcano maps for differentially expressed mRNAs in HAECs (A) and HUVECs of GSE103672 (Ajami et al., 2017) (B) with FoldChange≥2 (*P*<0.05). Blue, downregulated expression; gray, no differential expression; red, upregulated expression. Heat map for downregulated mRNAs in HAECs (C) and HUVECs of GSE103672 (Ajami et al., 2017) (D) compared with the static group. Red, high expression; blue, low expression. Results are from three replicates. (E) The flow chart of screening for the 25 key mRNAs from the high-throughput sequencing data of HAECs and HUVECs of GSE103672 (Ajami et al., 2017). Gene Ontology function enrichment analysis of 25 key mRNAs in biological processes (F), cellular components (G), and molecular functions (H). Bar chart (I) and bubble chart (J) of the KEGG pathway enrichment analysis of the 25 key mRNAs (K,L). Based on the results of the STRING database analysis (unconnected nodes were removed), the PPI network analysis was performed on key mRNAs. (M) The mRNA expression of AS-related mRNAs in static and Lss-treated HAECs was determined by qRT-PCR. (N) The mRNA expression of *CX3CL1* in static and Lss-treated HUVECs. The protein expression of CX3CL1 in the supernatant and inside HAECs was determined by ELISA (O) and western blotting (P,Q), respectively. The results are presented as mean±s.e.m. of three independent experiments. *P<0.05, **P<0.01, ***P<0.001 compared with the static group (unpaired two-tailed *t*-test).

To identify Lss-sensitive mRNAs in human aortic endothelial cells (HAECs), we performed whole-transcriptome sequencing on static HAECs (HAECs-static group) and HAECs exposed to Lss (HAEC-Lss group). We then used the edgR software package of R language to identify differences in the sequencing data between the two groups [| log2FoldChange | >1, false discovery rate (FDR)<0.05]. The results showed that compared with the HAECs-static group, the expression of 452 mRNAs was significantly changed in the HAECs-Lss group (Fig. 1A). Specifically, 297 mRNAs were upregulated, whereas 155 mRNAs were downregulated (Fig. 1C; Table S1). In addition, we analyzed previous high-throughput sequencing data (GSE103672; Ajami et al., 2017) to identify differences between static human umbilical vein endothelial cells (HUVECs; HUVECs-static group) and Lss-treated HUVECs (HUVECs-Lss group). The results showed that compared with the HUVECs-static group, the expression of 1349 mRNAs was significantly changed in the HUVECs-Lss group (Fig. 1B). Specifically, 496 mRNAs were upregulated, whereas 853 mRNAs were downregulated (Fig. 1D; Table S2). To explore the effect of Lss on the function of vascular endothelial cells, we identified 25 mRNAs that were downregulated in both Lss-treated HAECs and HUVECs as key mRNAs (Fig. 1E; Table 1). Gene Ontology function enrichment and Kyoto Encyclopedia of Genes and Genomes (KEGG) pathway enrichment analyses showed that the biological functions and pathways enriched by these 25 key mRNAs were very important in the occurrence and development of AS, such as regulation of cell adhesion, macrophage chemotaxis, CSF1–CSF1R complex, CX3C chemokine receptor binding, TGF-β signaling pathway, chemokine signaling pathway and TNF signaling pathway (Fig. 1F–J). To further explore the functions of these 25 key mRNAs at the protein level, based on the results of the STRING database, we established a protein–protein interaction (PPI) network with these 25 key mRNAs, which consisted of 21 nodes and 36 edges (Fig. 1K,L). Studies have reported that CXCR4, APLN, CX3CL1, CSF1 and HES1 are involved in the occurrence and development of AS (Döring et al., 2017; Gunter et al., 2017; Li et al., 2021; Lin et al., 2018). Therefore, this study used quantitative real-time PCR (qRT-PCR) to measure the mRNA expression of *CXCR4*, *APLN*, *CX3CL1*, *CSF1* and *HES1* in static and Lss-treated HAECs. As shown in Fig. 1M, compared with the HAECs-static group, the relative mRNA expression of *CX3CL1*, *CSF1* and *HES1* was reduced in the HAECs-Lss group, which is consistent with the results of the above-mentioned biosynthesis analysis. In view of the most significant reduction in *CX3CL1* mRNA expression, we identified *CX3CL1* as a target gene for follow-up studies. As shown in Fig. 1N, compared with the HUVECs-static group, the mRNA expression of *CX3CL1* was also reduced in the HUVECs-Lss group, which is in line with the results of the above-mentioned biosynthesis analysis. Moreover, compared with the static group, the expression of proteins in the supernatant and inside HAECs in the HAECs-Lss group was reduced (Fig. 1O–Q). The above results indicate that Lss can reduce *CX3CL1* mRNA and protein expression in endothelial cells, so *CX3CL1* is a Lss-sensitive gene.

**Table 1.**
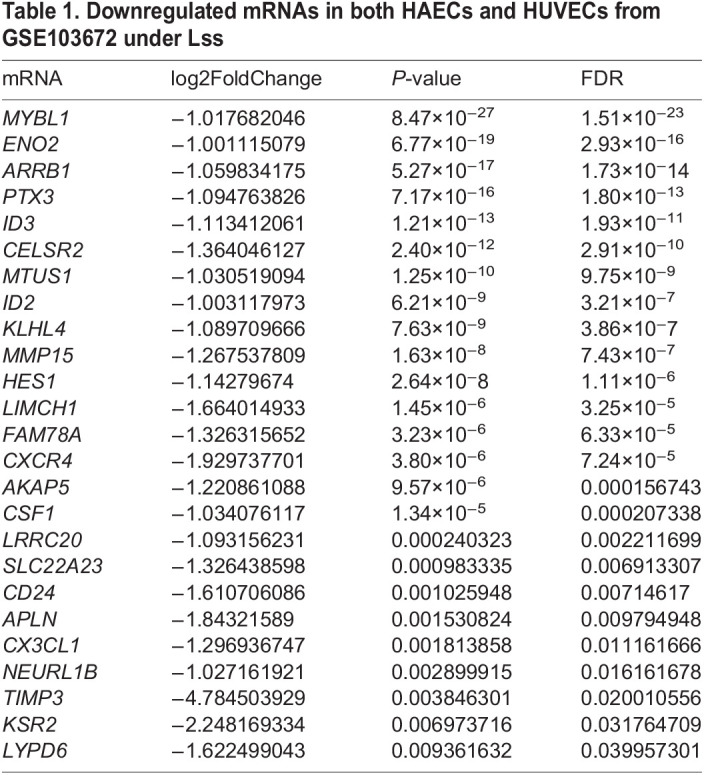
Downregulated mRNAs in both HAECs and HUVECs from GSE103672 under Lss

### Lss impairs monocyte adhesion to activated HAECs by inhibiting CX3CL1 expression

**Fig. 2.**
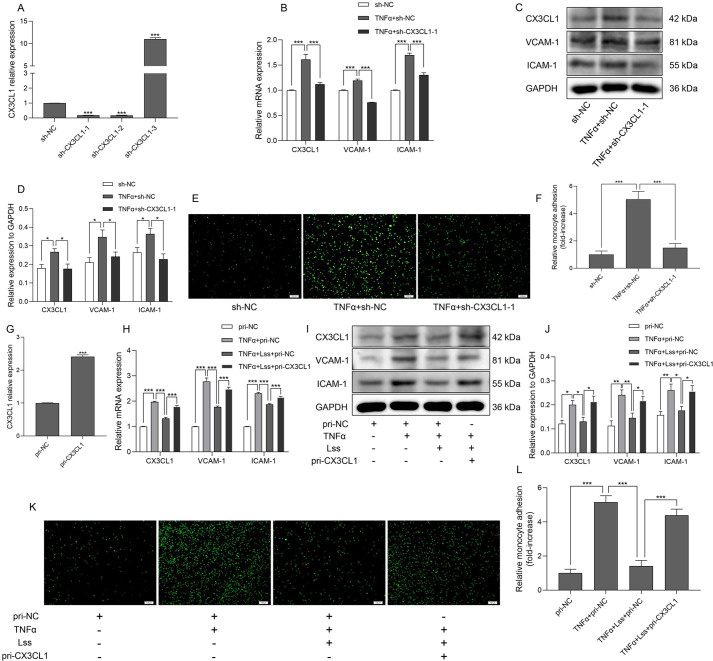
**Lss impairs monocyte adhesion to activated HAECs by inhibiting CX3CL1 expression.** (A) After HAECs were transfected with sh-CX3CL1-1, -2, -3, or sh-NC, *CX3CL1* expression was determined by qRT-PCR. The mRNA (B) and protein (C,D) expression of CX3CL1, VCAM-1 and ICAM-1 with and without CX3CL1 knockdown in TNF-α-treated and non-treated HAECs. (E,F) Monocyte adhesion to HAECs was determined using the monocyte adhesion assay. (G) After HAECs were transfected with pri-CX3CL1 or pri-NC, CX3CL1 expression was determined by qRT-PCR. HAECs were transfected with pri-CX3CL1 or pri-NC in the presence or absence of TNF-α (10 ng/ml) and then selectively subjected to Lss for 12 h. The mRNA (H) and protein (I,J) expression of CX3CL1, VCAM-1 and ICAM-1 were determined by qRT-PCR and western blotting, respectively. (K,L) Monocyte adhesion to HAECs was determined using the monocyte adhesion assay. The results are presented as mean±s.e.m. of three independent experiments. *P<0.05, **P<0.01, ***P<0.001 (one-way ANOVA followed by a Tukey's post-hoc test). Scale bars: 100 µm.

To explore the effect of CX3CL1 on the biological functions of HAECs, we established a cell model of CX3CL1 knockdown by transfecting HAECs with short hairpin RNAs (shRNAs). As shown in Fig. 2A, sh-CX3CL1-1 had the highest knockdown efficiency, so it was selected for subsequent research. Although not used further, we are unsure why sh-CX3CL1-3 led to a drastic increase in expression.

Studies have reported that endothelial CX3CL1 induces monocyte adhesion, thereby damaging vascular endothelial function (Babendreyer et al., 2017). qRT-PCR and western blotting showed that TNF-α induced an increase in the mRNA and protein expression of CX3CL1, vascular cell adhesion molecule-1 (VCAM-1), and intercellular adhesion molecule-1 (ICAM-1) in HAECs compared with the negative control group. However, the expression of CX3CL1, VCAM-1, and ICAM-1 was inhibited after transfection of sh-CX3CL1-1 (Fig. 2B–D). In addition, reduced CX3CL1 expression inhibited the enhancement of TNF-α-stimulated monocyte adhesion (Fig. 2E,F).

We further explored whether Lss affects endothelial cell function by regulating CX3CL1 expression. After HAECs were transfected with the CX3CL1 overexpression plasmid (pri-CX3CL1), the mRNA expression of CX3CL1 was significantly upregulated, indicating that the CX3CL1 overexpression cell model was successfully established (Fig. 2G).

As shown in Fig. 2H–J, Lss significantly inhibited the increase in TNF-α-stimulated CX3CL1, VCAM-1, and ICAM-1 mRNA and protein expression, and upregulating the expression of CX3CL1 reversed the inhibitory effect of Lss. In addition, an analysis of monocyte adhesion showed that overexpression of CX3CL1 partially reversed the inhibitory effect of Lss on the enhancement of TNF-α-stimulated monocyte adhesion (Fig. 2K,L). The above results indicate that Lss impairs monocyte adhesion to activated HAECs by inhibiting CX3CL1 expression.

### CX3CL1 is a direct target of miR-29b-3p

**Fig. 3.**
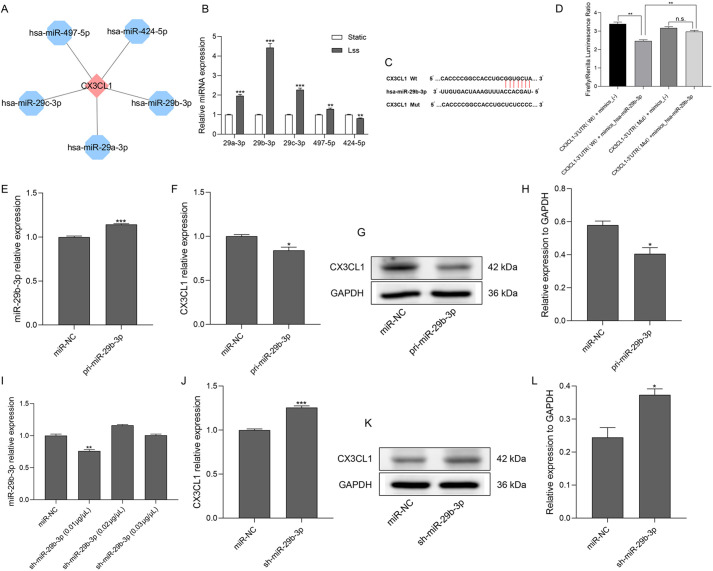
**CX3CL1 is a direct target of miR-29b-3p.** (A) Five upstream miRNAs with conservative binding sites to CX3CL1 were predicted using the TargetScan and DIANA TOOLS databases. (B) The expression of five key miRNAs in static and Lss-treated HAECs was determined by qRT-PCR. (C,D) The luciferase reporter gene vector containing CX3CL1-3'UTR Wt or Mut was co-transfected into 293T cells with mimics_hsa-miR-29b-3p or negative control to observe the targeting relationship between CX3CL1 and miR-29b-3p. (E) After HAECs were transfected with pri-miR-29b-3p or negative control, miR-29b-3p expression was determined by qRT-PCR. The mRNA (F) and protein (G,H) expression of CX3CL1 with or without miR-29b-3p overexpression is shown. (I) After HAECs were transfected with sh-miR-29b-3p (0.01, 0.02 or 0.03 μg/μl) or negative control, miR-29b-3p expression was determined by qRT-PCR. The mRNA (J) and protein (K,L) expression of *CX3CL1* with or without miR-29b-3p knockdown is shown. The results are presented as mean±s.e.m. of three independent experiments. *P<0.05, **P<0.01, ***P<0.001 (unpaired two-tailed *t*-test for two group comparison, and one-way ANOVA followed by a Tukey’s post-hoc test for three or more comparisons).

miRNA expression is very sensitive to fluid shear stress (Mai et al., 2013). Recent studies have reported that miRNAs regulated by fluid shear stress are potential targets for the treatment of cardiovascular diseases (Zhang et al., 2021). Therefore, to analyze the molecular mechanism of Lss-dependent protection of endothelial cell function, this study examined upstream miRNAs that regulate CX3CL1 expression in response to Lss. Based on the TargetScan and DIANA TOOLS databases, five miRNAs (miR-424-5p, miR-497-5p, miR-29c-3p, miR-29a-3p, and miR-29b-3p) were predicted to be upstream miRNAs with conserved binding sites to CX3CL1 (Fig. 3A, Table 2). qRT-PCR showed that compared with the HAECs-static group, Lss most significantly upregulated the expression of miR-29b-3p (Fig. 3B). To further confirm the interaction between miR-29b-3p and CX3CL1, we constructed a luciferase reporter gene vector (pmirGLO Vector-CX3CL1-3′UTR) of CX3CL1-3′UTR wild type (Wt) and mutant (Mut) (Fig. 3C), as well as mimics_hsa-miR-29b-3p and negative control mimics [mimics_(-)]. As shown in Fig. 3D, overexpression of miR-29b-3p significantly inhibited luciferase activity in the CX3CL1-3′UTR Wt group, but this effect was eliminated after pmirGLO Vector-CX3CL1-3′UTR (Mut) transfection. After HAECs were transfected with a plasmid overexpressing miR-29b-3p (pri-miR-29b-3p) or a plasmid with miR-29b-3p knockdown [sh-miR-29b-3p (0.01 µg/µl)], miR-29b-3p expression was upregulated or downregulated accordingly, indicating that the cell models of miR-29b-3p overexpression or knockdown were successfully constructed (Fig. 3E,I). Further verification showed that overexpression of miR-29b-3p inhibited CX3CL1 mRNA and protein expression, whereas miR-29b-3p knockdown promoted CX3CL1 mRNA and protein expression (Fig. 3F–H,J–L). The above results indicate that CX3CL1 is a direct target of miR-29b-3p.

**Table 2.**
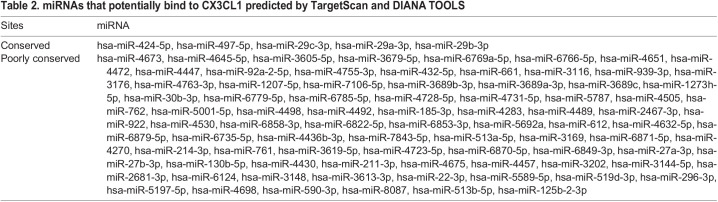
miRNAs that potentially bind to CX3CL1 predicted by TargetScan and DIANA TOOLS

### Lss impairs monocyte adhesion to activated HAECs via regulation of the miR-29b-3p/CX3CL1 axis

**Fig. 4.**
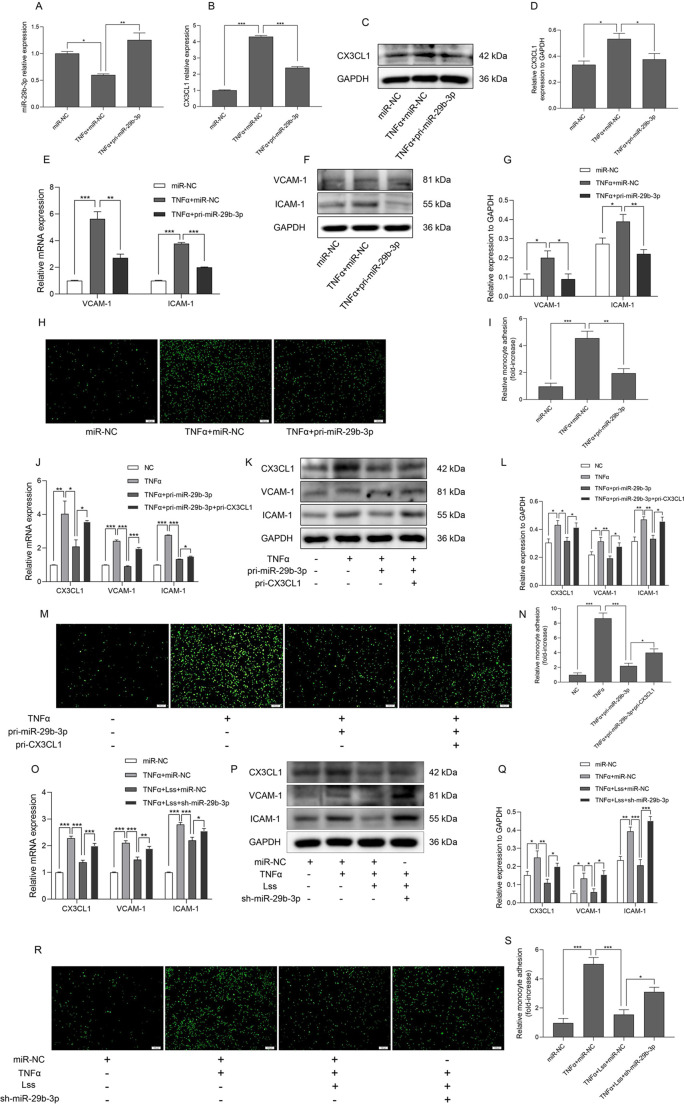
**Lss impairs monocyte adhesion to activated HAECs through regulating the miR-29b-3p/CX3CL1 axis.** HAECs were transfected with pri-miR-29b-3p or negative control in the presence or absence of TNF-α (10 ng/ml). (A) miR-29b-3p expression was determined by qRT-PCR. The mRNA (B) and protein (C,D) expression of CX3CL1 were determined by qRT-PCR and western blotting, respectively. The mRNA (E) and protein (F,G) expression of VCAM-1 and ICAM-1. (H,I) Monocyte adhesion to HAECs was determined using the monocyte adhesion assay. In TNF-α-treated HAECs, pri-miR-29b-3p was transfected into cells in the presence or absence of pri-CX3CL1. The mRNA (J) and protein (K,L) expression of CX3CL1, VCAM-1 and ICAM-1 is shown. (M,N) Monocyte adhesion to HAECs was determined using the monocyte adhesion assay. HAECs were transfected with sh-miR-29b-3p or negative control in the presence or absence of TNF-α (10 ng/ml), and were then selectively subjected to Lss for 12 h. The mRNA (O) and protein (P,Q) expression of CX3CL1, VCAM-1 and ICAM-1 were determined by qRT-PCR and western blotting, respectively. (R,S) Monocyte adhesion to HAECs was determined using the monocyte adhesion assay. The results are presented as mean±s.e.m. of three independent experiments. *P<0.05, **P<0.01, ***P<0.001 (one-way ANOVA followed by a Tukey's post-hoc test). Scale bars: 100 µm.

As shown in Fig. 4A, compared with the negative control group, TNF-α induced downregulation of miR-29b-3p expression in HAECs, while transfection of pri-miR-29b-3p restored miR-29b-3p expression, indicating that miR-29b-3p was successfully overexpressed in TNF-α-activated HAECs.

Subsequently, this study explored whether miR-29b-3p regulates the expression of CX3CL1 in TNF-α-activated HAECs. qRT-PCR and western blotting showed that miR-29b-3p overexpression significantly inhibited TNF-α-induced elevation of CX3CL1 mRNA and protein expression (Fig. 4B–D). In addition, miR-29b-3p overexpression also significantly inhibited the TNF-α-induced increase in VCAM-1 and ICAM-1 mRNA and protein expression, as well as the enhancement of monocyte adhesion (Fig. 4E–I). To further verify whether miR-29b-3p inhibited monocyte adhesion to activated HAECs by regulating CX3CL1 expression, pri-miR-29b-3p and pri-CX3CL1 were co-transfected into TNF-α-activated HAECs. As shown in Fig. 4J–L, in TNF-α-activated HAECs, overexpression of CX3CL1 eliminated the inhibitory effect of miR-29b-3p on CX3CL1, VCAM-1 and ICAM-1 mRNA and protein expression. In addition, miR-29b-3p overexpression significantly inhibited monocyte adhesion to TNF-α-activated HAECs, while CX3CL1 overexpression reversed this inhibition (Fig. 4M,N). Given that miR-29b-3p is a Lss-sensitive miRNA, we also explored the effect of miR-29b-3p knockdown on endothelial cell adhesion under the condition of loading Lss. As shown in Fig. 4O–Q, the mRNA and protein expression of CX3CL1, VCAM-1 and ICAM-1 upregulated by TNF-α were inhibited after loading Lss, while knockdown of miR-29b-3p reversed this inhibition. Similarly, miR-29b-3p knockdown also reversed the inhibitory effect of Lss on monocyte adhesion to TNF-α-activated HAECs (Fig. 4R,S). The above results indicate that Lss impairs monocyte adhesion to activated HAECs through regulation of the miR-29b-3p/CX3CL1 axis.

### The Lss-sensitive miR-29b-3p/CX3CL1 axis alleviates inflammation by blocking NF-κB signaling in HAECs

**Fig. 5.**
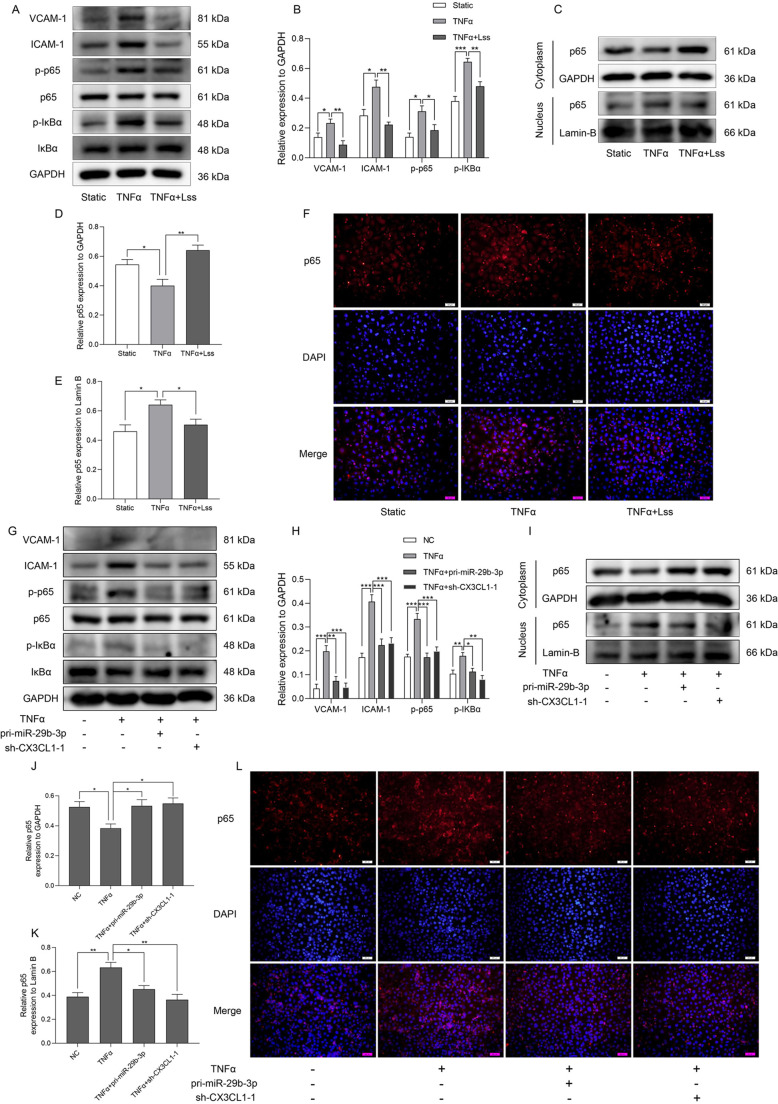
**The Lss-sensitive miR-29b-3p/CX3CL1 axis alleviates inflammation through blocking the NF-κB signaling pathway in HAECs.** HAECs were subjected to Lss for 12 h in the presence or absence of TNF-α (10 ng/ml). (A,B) VCAM-1, ICAM-1, phosphorylated (p)-p65, p65, p-IκBα, and IκBα protein expression were determined by western blotting. (C–E) The protein expression of p65 in the cytoplasm and nucleus of HAECs is shown. (F) The expression and localization of p65 in HAECs were determined by immunofluorescence. HAECs were transfected with pri-miR-29b-3p or sh-CX3CL1 in the presence or absence of TNF-α (10 ng/ml). (G,H) VCAM-1, ICAM-1, p-p65, p65, p-IκBα, and IκBα protein expression was determined by western blotting. (I–K) The protein expression of p65 in the cytoplasm and nucleus of HAECs is shown. (L) The expression and localization of p65 in HAECs were determined by immunofluorescence. The results are presented as mean±s.e.m. of three independent experiments. *P<0.05, **P<0.01, ***P<0.001 (one-way ANOVA followed by a Tukey's post-hoc test). Scale bars: 50 µm.

The NF-κB signaling pathway is very important in the regulation of inflammatory diseases. Zhong et al. proposed that some endothelial miRNAs control the transcription of cell adhesion molecules by regulating NF-κB signaling, thereby affecting monocyte adhesion to endothelial cells (Zhong et al., 2018). To further clarify the molecular mechanism of Lss-sensitive miR-29b-3p/CX3CL1 axis-mediated regulation of endothelial cell adhesion, we analyzed the effects of Lss, miR-29b-3p and CX3CL1 on the NF-κB signaling pathway in TNF-α-activated HAECs. As shown in Fig. 5A,B, after loading Lss, the upregulation of VCAM-1 and ICAM-1 induced by TNF-α was inhibited. In addition, Lss significantly inhibited p65 and IκBα (also known as RELA and NFKBIA, respectively) phosphorylation induced by TNF-α (Fig. 5A,B), indicating that Lss might regulate endothelial cell inflammation by inhibiting the activation of NF-κB signaling. Nuclear translocation of p65 is the key to activation of NF-κB signaling. As shown in Fig. 5C–E, after loading Lss, the accumulation of p65 in the nuclei of TNF-α-activated HAECs was significantly reduced. Furthermore, immunofluorescence analysis showed that Lss significantly reduced TNF-α-induced nuclear translocation of p65 (Fig. 5F). Notably, miR-29b-3p knockdown reversed the inhibitory effect of Lss on the NF-κB signaling pathway in TNF-α-activated HAECs (Fig. S5D,E). We also explored whether the Lss-sensitive miR-29b-3p/CX3CL1 axis could regulate NF-κB signaling in TNF-α-activated HAECs (Fig. S2D,E). As shown in Fig. 5G,H and Fig. S4, overexpression of miR-29b-3p significantly inhibited the upregulation of TNF-α-stimulated VCAM-1 and ICAM-1, as well as the phosphorylation of p65 and IκBα. The same results were obtained in TNF-α-activated HAECs with CX3CL1 knockdown. Overexpression of miR-29b-3p or knockdown of CX3CL1 reduced the accumulation of p65 in the nuclei of TNF-α-activated HAECs (Fig. 5I–K). Moreover, immunofluorescence analysis also showed that overexpression of miR-29b-3p or knockdown of CX3CL1 substantially reduced TNF-α-induced nuclear translocation of p65 (Fig. 5L). The above results indicate that the Lss-sensitive miR-29b-3p/CX3CL1 axis alleviates endothelial cell inflammation by blocking the NF-κB signaling pathway.

### miR-29b-3p alleviates HFD-induced endothelial inflammation and AS in *ApoE*^−/−^ mice

In order to link Lss with miR-29b-3p and CX3CL1 *in vivo*, we investigated the expression levels of miR-29b-3p and CX3CL1 in the intima of the aortic arch of *ApoE*^−/−^ mice exposed to turbulence (Oss) and the intima of the descending thoracic aorta exposed to Lss (Fig. S3A). As shown in Fig. S3B,C, the expression of miR-29b-3p in the thoracic aortic intima exposed to Lss was upregulated, whereas the expression of CX3CL1 was downregulated compared with the aortic arch. Moreover, *in vitro* studies found that the expression of CX3CL1 in HAECs treated with oscillatory shear stress (0.5±4 dyn/cm^2^) was upregulated, while the expression of miR-29b-3p was downregulated (Fig. S5A–C).

**Fig. 6.**
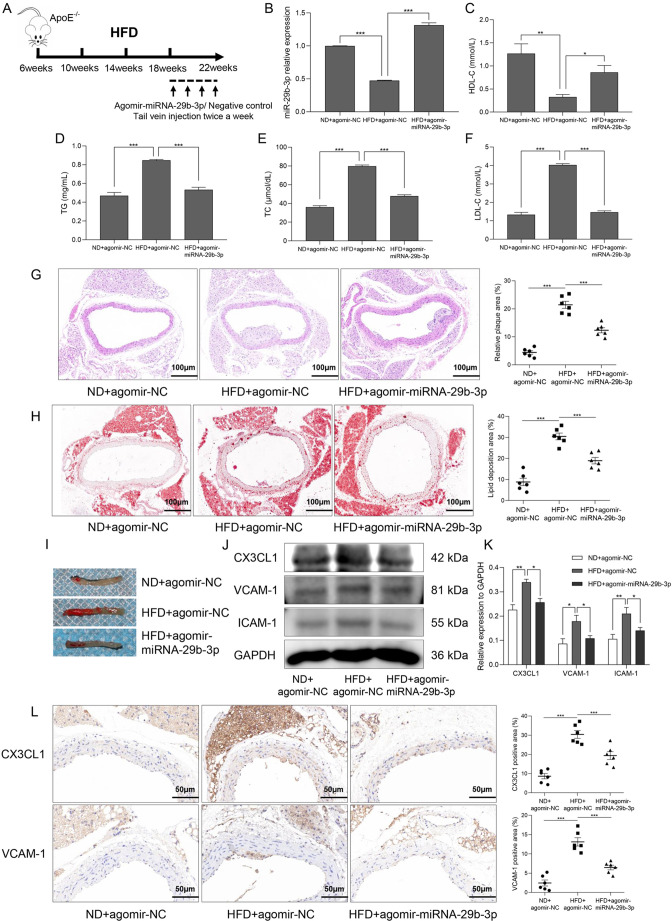
**MiR-29b-3p alleviates HFD-induced endothelial inflammation and AS in *ApoE*^−/−^ mice.** (A) The process used to establish the mouse model of AS. (B) miR-29b-3p expression in aortic intima was determined by qRT-PCR. The expression of HDL-C (C), TG (D), TC (E), and LDL-C (F) in the serum of *ApoE*^−/−^ mice was measured using biochemical detection kits. (G) Pathological changes in the aorta were determined by Hematoxylin and Eosin staining. (H,I) Lipid deposition in the aorta was determined by Oil Red O staining. (J,K) The protein expression of CX3CL1, VCAM-1, and ICAM-1 in the aortic intima was determined by western blotting. (L) The expression of CX3CL1 and VCAM-1 in the aorta was determined by immunohistochemistry. There were six mice per group, and the results are presented as mean±s.e.m. *P<0.05, **P<0.01, ***P<0.001 (one-way ANOVA followed by a Tukey's post-hoc test).

To confirm whether miR-29b-3p regulates AS progression *in vivo*, male *ApoE*^−/−^ mice aged 6 weeks were fed with either a normal diet (ND) or an HFD for 4 months. Agomir-miR-29b-3p or negative control was injected during the last month of the feeding cycle (Fig. 6A). qRT-PCR showed that compared with *ApoE*^−/−^ mice fed the ND, miR-29b-3p expression in aortic intima from *ApoE*^−/−^ mice fed the HFD was significantly reduced. However, a tail vein injection of agomir-miRNA-29b-3p restored the expression of miR-29b-3p in aortic intima (Fig. 6B). In addition, agomir-miRNA-29b-3p could also inhibit the mRNA and protein expression levels of CX3CL1 *in vitro* (Fig. S2F–H). Subsequently, this study used biochemical detection kits to measure the concentrations of high-density lipoprotein cholesterol (HDL-C), triglyceride (TG), total cholesterol (TC), and low-density lipoprotein cholesterol (LDL-C) in serum from *ApoE*^−/−^ mice. The results showed that, compared with *ApoE*^−/−^ mice fed the ND, the concentration of HDL-C in serum from *ApoE*^−/−^ mice fed the HFD was significantly reduced, whereas the concentrations of TG, TC and LDL-C were significantly increased. However, when miR-29b-3p was overexpressed, the concentrations of HDL-C, TG, TC, and LDL-C were restored (Fig. 6C–F). As shown in Fig. 6G, compared with *ApoE*^−/−^ mice fed the ND, the intimal surface of the aorta in *ApoE*^−/−^ mice fed the HFD was not smooth. This was accompanied by intimal thickening and larger plaque formation, indicating that the AS mouse model was successfully established. However, when agomir-miRNA-29b-3p was injected, the plaque area of the aortic intima in *ApoE*^−/−^ mice fed the HFD was significantly reduced (Fig. 6G). In addition, Oil Red O staining also showed that overexpression of miR-29b-3p partially alleviated HFD-induced lipid deposition in the aortic intima of *ApoE*^−/−^ mice (Fig. 6H,I). The above results indicate that miR-29b-3p improves AS *in vivo*.

We also explored whether miR-29b-3p regulates intimal inflammation in mice with AS by controlling the expression of CX3CL1, thereby affecting the occurrence and development of AS. Western blotting showed that overexpression of miR-29b-3p reversed the increase in CX3CL1, VCAM-1 and ICAM-1 protein expression in aortic intima from *ApoE*^−/−^ mice fed the HFD (Fig. 6J,K). Moreover, immunohistochemical staining showed that compared with ApoE^−/−^ mice fed the ND, the expression of CX3CL1 and VCAM-1 in the aorta of *ApoE*^−/−^ mice fed the HFD was significantly increased. However, overexpression of miR-29b-3p downregulated the expression of CX3CL1 and VCAM-1 in the aorta (Fig. 6L). In short, miR-29b-3p alleviated endothelial cell inflammation *in vivo*, thereby improving AS.

## DISCUSSION

AS is an important cause of cardiovascular diseases, such as coronary heart disease, myocardial infarction and peripheral vascular disease, and the incidence of AS is high. AS is related to a variety of stimulating factors, such as high blood pressure, diabetes mellitus, smoking, chronic inflammation, a HFD and genetic factors, among which vascular inflammation is the key inducer of AS (Ross, 1999). When these stimulating factors damage the vascular endothelium, E-selectin, VCAM-1 and ICAM-1 are upregulated in the endothelium by activating the transcription messenger NF-κB, thereby inducing monocytes to adhere to the vascular intima (Raggi et al., 2018). As they penetrate into the lower inner membrane, monocytes gradually differentiate into macrophages, phagocytose the lipids deposited around them, and develop into foam cells. The foam cells further recruit inflammatory factors and chemokines, leading to local inflammation and plaque formation (Hansson et al., 2006). Therefore, alleviating vascular inflammation is an effective strategy to prevent and treat AS. Studies have found that knocking down nucleophosmin in *ApoE*^−/−^ mice fed a HFD can improve vascular inflammation, reduce macrophage infiltration, and stabilize endothelial cell function, thereby reducing the pathological change in AS and promoting plaque stability (Rao et al., 2021). Annexin A5 can also improve vascular function by mediating vascular inflammation and vascular remodeling, thereby slowing the progression of AS (Ewing et al., 2011). Through *in vivo* and *in vitro* studies, Zhang et al. found that irisin can reduce oxidized LDL-induced inflammation and apoptosis of vascular endothelial cells, thus playing an anti-AS role (Zhang et al., 2016). In addition, fluid shear stress can influence AS by regulating vascular endothelial cell inflammation. Previous studies from our group have shown that Lss regulates the inflammatory response in HUVECs by mediating the AF131217.1/miR-128-3p/KLF4 axis, thereby affecting the occurrence and development of AS (Lu et al., 2019). Another study found that Lss regulated the expression of lectin-like oxidized low-density lipoprotein receptor-1 (LOX-1) in endothelial cells by mediating the KLF2/AP1 pathway, thereby affecting endothelial cell function and AS formation (Lee et al., 2018). Doddaballapur et al. proposed that Lss reduced the expression of the key glycolytic enzyme PFKFB3 in a KLF2-dependent manner, thereby inhibiting endothelial cell metabolism and regulating endothelial cell phenotype (Doddaballapur et al., 2015). In addition, studies have found that Lss can upregulate the expression of miR-30-5p family by mediating KLF2 to attenuate the expression of Ang2 and adhesion molecules, thereby inhibiting endothelial cell inflammation (Demolli et al., 2015). This study found that the Lss-sensitive miR-29b-3p/CX3CL1 axis alleviates monocyte adhesion to TNF-α-activated HAECs by inhibiting the activation of NF-κB signaling, thereby maintaining endothelial cell function and reducing the occurrence of AS. In addition, in order to further clarify the molecular mechanism of CX3CL1 regulating endothelial cell inflammation, this study continued to explore whether the CX3CR1 signaling pathway is involved in the inflammatory regulation mechanism of CX3CL1. Small interfering RNA (si-CX3CR1) was used to inhibit the expression of CX3CR1 in HAECs (Fig. S2A). As shown in Fig. S2B,C, decreased expression of CX3CR1 significantly inhibited TNF-α-stimulated upregulation of VCAM-1 and ICAM-1 expression, as well as the activation of NF-κB signaling pathway. Therefore, CX3CL1 might regulate endothelial cell inflammation through an autocrine amplification loop induced by the CX3CL1/CX3CR1 axis.

Chemokines are a type of cytokine secreted by cells that can induce targeted chemotaxis of nearby cells. According to the sequence of N-terminal cysteine residues, they can be divided into four subfamilies: CXC, CC, CX3C and XC (Miller and Mayo, 2017). Chemokines are not only expressed in blood vessel walls, but also in migrating leukocytes, so they are closely related to the occurrence and development of AS (Zernecke and Weber, 2014). Lysophosphatidic acid (LPA) derived from oxidized LDL in the blood vessel wall triggers the deposition of CXCL1 in the vascular endothelium, thereby inducing monocyte migration and adhesion, which is a key process in the early stages of AS (Zhou et al., 2011). In addition, CCL5 plays a key role in monocyte recruitment and macrophage abundance in the early stage of AS (Jongstra-Bilen et al., 2021). Chemokines not only recruit monocytes to the intima of blood vessels, but they also participate in the regulation of endothelial cell dysfunction, macrophage differentiation, foam cell formation and other AS development processes. Woller et al. found that monocytes activated by CXCL4 induce vascular endothelial cell apoptosis by releasing a large amount of reactive oxygen species, thereby damaging endothelial function (Woller et al., 2008). CXCL8 can affect foam cell formation by inhibiting ABCA1 expression and cholesterol efflux (Tang et al., 2019). CX3CL1 plays an important role in AS development due to its dual functions in chemotaxis and adhesion. Human aortic smooth muscle cells (SMCs) exposed to a high concentration of glucose increase monocyte–SMC adhesion interactions by upregulating CX3CL1 and MCP-1 expression, thereby exacerbating vascular inflammation in patients with diabetes mellitus (Dragomir et al., 2008). Chang et al. found that tanshinone IIA downregulates the expression of VCAM-1, ICAM-1 and CX3CL1 by inhibiting the activation of the IKK/NF-κB signaling pathway in TNF-α-stimulated HUVECs, thereby destroying the adhesion of monocytes to activated HUVECs (Chang et al., 2014). In addition, CX3CL1 expression is significantly elevated in aortic tissues from *ApoE*^−/−^ mice fed a HFD (Liu et al., 2010). A recent study found that *in vitro* co-culture of CD16+ monocytes with high expression of CX3CR1 and primary endothelial cells could promote the formation of the pro-AS endothelial cell phenotype, which is mainly achieved by the CX3CR1–CX3CL1 interaction (Roy-Chowdhury et al., 2021). This present study focused on the adhesion role of CX3CL1 and revealed the potential mechanism of Lss on CX3CL1 to exert an anti-AS effect. Bazan et al. proposed that the full-length molecule of CX3CL1 is 95 kDa (Bazan et al., 1997); however, the molecular mass of CX3CL1 detected in this study is located at 42 kDa (Fig. S2I), which might be due to the selection of a specific antibody to detect a specific cleavage product.

As a member of the miR-29 family, miR-29b-3p is an miRNA that plays a role in the occurrence and development of a variety of diseases. Inhibition of miR-29b-3p expression can reduce the cell viability of breast cancer cells and inhibit cell migration and invasion (Zhang et al., 2019). In addition, miR-29b-3p gene promoter methylation can promote angiogenesis, invasion and migration of pancreatic cancer cells (Wang et al., 2021). Therefore, miR-29b-3p is an important intervention target for tumors. Moreover, miR-29b-3p is a potential therapeutic target for osteoarthritis (OA). Chen et al. found that miR-29b-3p blocks the cell cycle progression of chondrocytes and induces apoptosis, whereas the injection of miR-29b-3p antagomir into the joint cavity improves cartilage loss from the knee joint in a rat OA model (Chen et al., 2017). Studies have also reported that miR-29b-3p inhibits cardiomyocyte proliferation by reducing NOTCH2 *in vitro* and induces zebrafish malformation, growth retardation, and cardiac dysfunction through embryonic injection (Yang et al., 2020). In addition, overexpression of miR-29b-3p significantly improves LPS-induced cardiac damage and dysfunction in mice (Li et al., 2020). Jiang et al. also proposed that miR-29b-3p improves vascular calcification by directly targeting MMP2 in rat vascular smooth muscle cells (Jiang et al., 2017). Therefore, miR-29b-3p also plays an important regulatory role in the occurrence and development of cardiovascular diseases. miR-29b-3p is also abundantly expressed in the liver and is closely related to the occurrence and development of liver fibrosis (Tao et al., 2018). There, it is possible that exogenous miR-29b-3p suppresses vascular inflammation and atherosclerosis through suppression of lipid metabolism in liver cells. In this study, we predicted that miR-29b-3p was an important regulator of the anti-AS effect of Lss using Lss-sensitive CX3CL1. We also confirmed that miR-29b-3p is a key target for destroying monocyte adhesion to activated HAECs and resisting the formation of AS *in vivo* and *in vitro*. In order to further clarify the regulatory effect of miR-29b-3p on endothelial cell inflammatory response, this study explored the effect of sh-miR-29b-3p on the inflammatory response of TNF-α-stimulated HAECs *in vitro*. As shown in Fig. S3D,E, the decreased expression of miR-29b-3p significantly aggravated the TNF-α-induced upregulation of the protein expression levels of VCAM-1 and ICAM-1. Moreover, sh-miR-29b-3p also showed a tendency to enhance the TNF-α-activated NF-κB signaling pathway, but there was no statistical difference.

In conclusion, the Lss-sensitive miR-29b-3p/CX3CL1 axis significantly inhibits monocyte adhesion to activated HAECs and alleviates local inflammation and plaque formation in *ApoE*^−/−^ mice fed a HFD. Therefore, it is a potential intervention target for the prevention and treatment of AS.

## MATERIALS AND METHODS

### Lss-treated cell model

The equipment for simulating Lss *in vitro* was purchased from Shanghai Naturethink Life & Scientific Co., Ltd. The equipment consisted of a peristaltic pump, a smoothing device, a flow chamber and a liquid storage bottle, connected by a silicone hose in a closed loop (Fig. S1). Cells were planted on a slide and cultured to a degree of 90% fusion. A cell-filled slide was then placed in the flow chamber, and the equipment was activated to apply 12 dyn/cm^2^ Lss to the cells in the chamber using the flow of culture medium. The Lss-treated cell model was obtained after 12 h of operating the equipment.

### Cell transfection

The HAEC and HUVEC cell lines were obtained from the Department of Pathogenobiology, Basic Medicine College of Jilin University (Changchun, China). The cell lines were cultured in DMEM or IMDM (Gibco, China) supplemented with 10% fetal bovine serum (Gibco, Australia) and 1% penicillin-streptomycin at 37°C with a 5% CO_2_ humidified atmosphere. All plasmids were purchased from Public Protein/Plasmid Library (Nanjing, China), including CX3CL1 overexpression plasmid pLVX-Puro-CX3CL1 (pri-CX3CL1), CX3CL1 knockdown plasmids pLKO.1-Puro-CX3CL1 shRNA (sh-CX3CL1-1, -2, and -3), miR-29b-3p overexpression plasmid pLenti-CMV-miR-29b-3p (pri-miR-29b-3p), miR-29b-3p sponge plasmid pLKO.1-miR-29b-3p sponge (sh-miR-29b-3p), and corresponding negative control plasmid. Relevant shRNA sequences are listed in Table S3. X-tremeGENE HP DNA Transfection Reagent (Roche, Basel, Switzerland) was used to transfect the above plasmids into HAECs. After 24 h of transfection, the cells were used for subsequent analysis.

### qRT-PCR

Total RNA was extracted from cells using miRcute miRNA Isolation Kit (Tiangen Biotech, China), and reverse transcription of mRNAs and miRNAs was performed using HiScript II 1st Strand cDNA Synthesis Kit (Vazyme, China) and miRcute miRNA First-strand cDNA Synthesis Kit (Tiangen Biotech, China), respectively. Then, the ABI 7300 Plus Real-Time PCR System (Applied Biosystems, USA) was run according to the standard protocol of FastStart Universal SYBR Green Master (Roche, Basel, Switzerland). When measuring the expression of miRNAs, the miRcute Plus miRNA qPCR kit (Tiangen Biotech, China) was used. All primer sequences used for qRT-PCR analysis are listed in Table S4, among which GAPDH and U6 were used as endogenous controls. The results were calculated using the 2^−ΔΔCt^ method.

### Western blotting

Total protein was extracted from cells with a Cell Protein Extraction Kit (Shanghai Epizyme Biomedical Technology, China), and the protein concentration was determined with the bicinchoninic acid protein detection kit (Beyotime Biotechnology, China). Total protein was separated by sodium dodecyl sulfate–polyacrylamide gel electrophoresis and transferred to polyvinylidene difluoride membrane. Antibodies against the following targets were added and incubated overnight at 4°C: CX3CL1 (Abcam, UK; ab85034, 1:900), VCAM-1 (bs-0396R, 1:1000), ICAM-1 (bs-6326R, 1:1000), GAPDH (bs-2188R, 1:5000), p65 (bs-23217R, 1:1000), p-p65 (bs-0982R, 1:1000), IκBα (bs-1287R, 1:1000), p-IκBα (bs-18128R, 1:1000) and lamin-B (bs-1840R, 1:1000) (all Bioss, Beijing, China). After incubating with horseradish peroxidase-conjugated AffiniPure goat anti-rabbit immunoglobulin G antibody, a chemiluminescence reaction was performed, and the intensity was analyzed with ImageJ software. Each western blot result shown is representative of three independent experiments. Please refer to the Blot Transparency section of supplementary information (Fig. S6) for images of all uncropped western blots in this study.

### Monocyte adhesion analysis

The THP-1 cell line was obtained from the Department of Pathogenobiology, Basic Medicine College of Jilin University (Changchun, China). THP-1 cells were labeled with CFSE (5- and 6-carboxyfluorescein diacetate succinimidyl ester; MedChemExpress, Monmouth Junction, NJ) at a final concentration of 5 μM and protected from light. The labeled THP-1 cells were incubated with HAECs for 4 h. Subsequently, serum-free 1640 medium (Meilunbio, China) was used to wash away the THP-1 cells that did not adhere. The adhesion of THP-1 cells to HAECs was observed under a fluorescence microscope (Olympus IX71, Japan), and the fluorescence intensity was analyzed with ImageJ software.

### Immunofluorescence

Cells grown on a slide were fixed with 4% paraformaldehyde for 30 min and then permeabilized with 0.5% Triton X-100 (Beyotime Biotechnology, China) for 15 min at room temperature. After incubating at room temperature for 30 min with goat serum (Boster Biotechnology, China), anti-NF-κB p65 antibody (cat. no. ab16502, Abcam) was added (2.5:1000) and incubated overnight at 4°C. After incubation with Cy3-labeled goat anti-rabbit IgG (H+L) (Beyotime Biotechnology, China) for 1 h, the nuclei were counterstained with DAPI (Beyotime Biotechnology, China) for 10 min. The slides were mounted, and the expression of p65 was observed under a fluorescence microscope (Olympus IX71, Japan).

### Luciferase reporter assay

The cDNA fragment containing the wild-type or mutant 3′ untranslated region of *CX3CL1* was subcloned downstream of the luciferase-encoding gene within the pmirGLO luciferase reporter vector (Promega, USA). The nucleotide sequences of all constructs were confirmed by DNA sequencing. Luciferase reporter plasmids plus mimics_hsa-miR-29b-3p or mimics_ (-) were co-transfected into 293T cells using Lipofectamine 2000 (Invitrogen, Carlsbad, CA, USA). After 48 h of transfection, a dual-luciferase reporter assay kit (MeilunBio, China) was utilized to measure luciferase activity.

### AS mouse model

*ApoE*^−/−^ mice (C57BL/6J background, 6 weeks of age, weighing 20–24 g) were purchased from Beijing Vital River Laboratory Animal Technology Co. Ltd. (Beijing, China), and raised in a specific pathogen-free environment. All animal experiments were performed in accordance with Jilin University Institutional Animal Care and Use Committee guidelines (Protocol No. 2015-34).

Mice were randomly divided into the ND group, the HFD group, and the HFD+agomir-miRNA-29b-3p group, with six mice in each group. ND (4% fat, 0% cholesterol) and HFD (20% fat, 1.25% cholesterol) foodstuffs were purchased from Synergy Pharmaceutical Bioengineering Co., Ltd. (Nanjing, China). *ApoE*^−/−^ mice were fed with the ND or the HFD for 4 months, and agomir-miRNA-29b-3p (50 mg/kg, Ribobio, China) or negative control was injected through the tail vein during the last month. After 4 months, blood was extracted, and the thoracic aorta was isolated for follow-up studies. After the aorta was cut with a scalpel, the subintimal space was found and the intima was peeled off with an intimal peeler and surgical instruments. The isolated aortic intima was used for qRT-PCR assay and western blotting assay.

### Histopathological analysis

The isolated aorta was soaked and fixed with 4% paraformaldehyde before being paraffin-embedded or frozen sectioned. Oil Red O and Hematoxylin and Eosin (H&E) staining were used to observe the lipid deposition and pathological changes of the aorta. To detect the expression of CX3CL1 and VCAM-1 in aortic tissue, frozen sections of aortic tissue were air-dried at room temperature and blocked with goat serum for 30 min. Then, anti-CX3CL1 antibody (Abcam; ab85034, 1:200) or anti-ICAM-1 antibody (Bioss, Beijing, China; bs-4618R, 1:200) was added in a dropwise fashion and incubated overnight at 4°C. The sections were cleaned with phosphate-buffered saline and then incubated with horseradish peroxidase-labeled secondary antibody at room temperature for 1 h. In the dark, 3,3′-diaminobenzidine chromogenic solution was added to the sections for color development, before counterstaining with hematoxylin. Then, the sections were mounted, and the expression of CX3CL1 and VCAM-1 was observed under a microscope.

### Statistical analysis

Data are expressed as mean±s.e.m., and all statistical analyses were conducted using SPSS 24.0 software. An unpaired two-tailed *t*-test was used for comparisons between two groups, and one-way analysis of variance (ANOVA) was used for comparisons between three or more groups, followed by a Tukey's post-hoc test. *P*<0.05 was considered statistically significant.

## Supplementary Material

Supplementary information

Reviewer comments
