# Supplementary material for: Laminar shear stress alleviates monocyte adhesion and atherosclerosis development via miR-29b-3p/CX3CL1 axis regulation
Source: J Cell Sci. 2022 Jul 22;135(14):jcs259696. doi: 10.1242/jcs.259696 (PMC9450891; doi:10.1242/jcs.259696)
Supplement: Supplementary information [file joces-135-259696-s1.pdf]

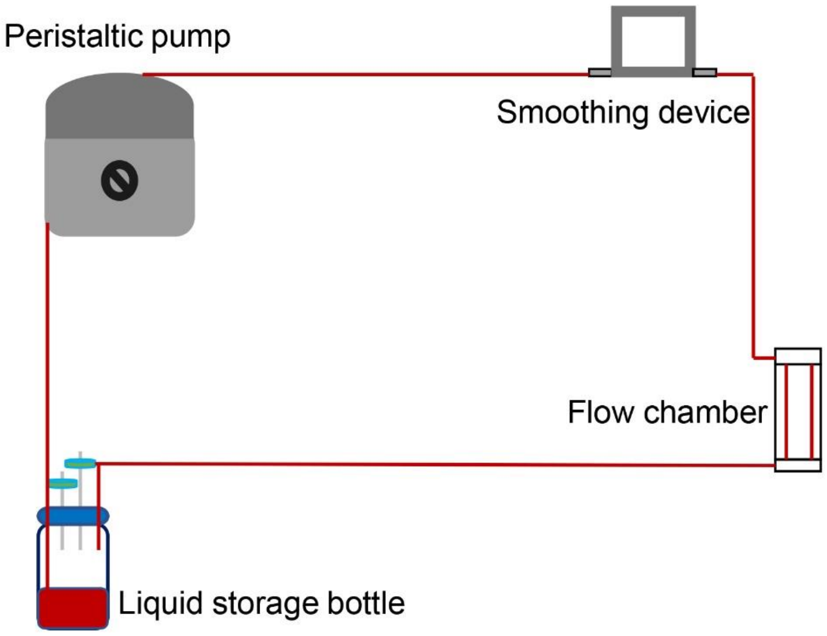

Fig. S1. Schematic diagram of the instrument used to simulate Lss in vitro.

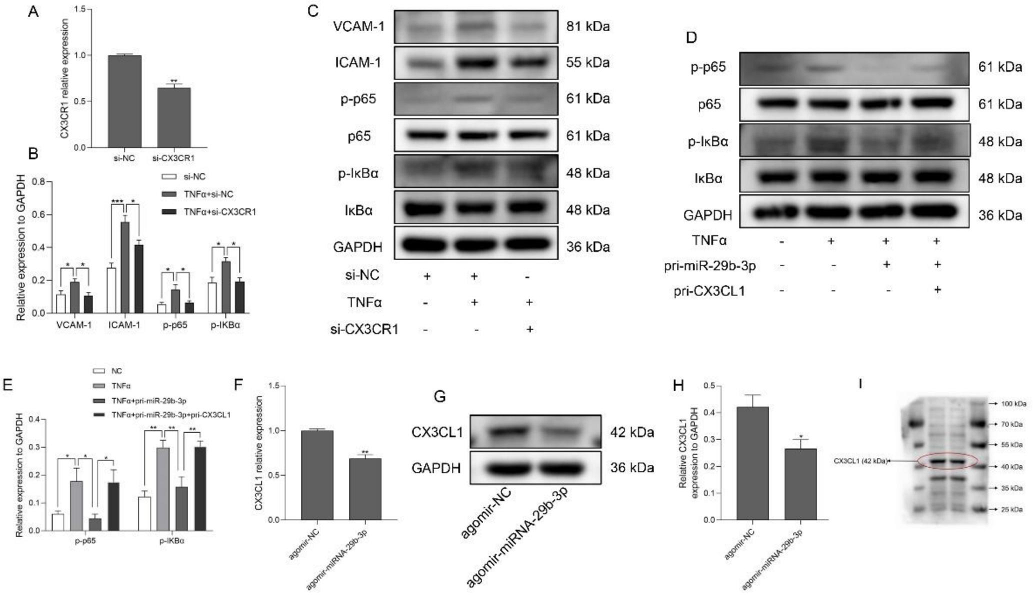

Fig. S2. (A) After HAECs were transfected with si-CX3CR1 or si-NC, CX3CR1 expression was determined by qRT-PCR. The protein (B and C) expression of VCAM-1, ICAM-1, p-p65, p65, p-IκBα and IκBα with/without CX3CR1 knockdown in TNF-

Journal of Cell Science • Supplementary information

α-treated and non-treated HAECs. In TNF-α-treated HAECs, pri-miR-29b-3p was transfected into cells in the presence or absence of pri-CX3CL1. The protein (D and E) expression of p-p65, p65, p-IκBα and IκBα. The mRNA (F) and protein (G and H) expression of CX3CL1 after HAECs were transfected with agomir-miRNA-29b-3p or agomir-NC. (I) The whole western blot showing the position of CX3CL1. The results are presented as mean ± SEM of three independent experiments. *$p < .05$, **$p < .01$, ***$p < .001$.

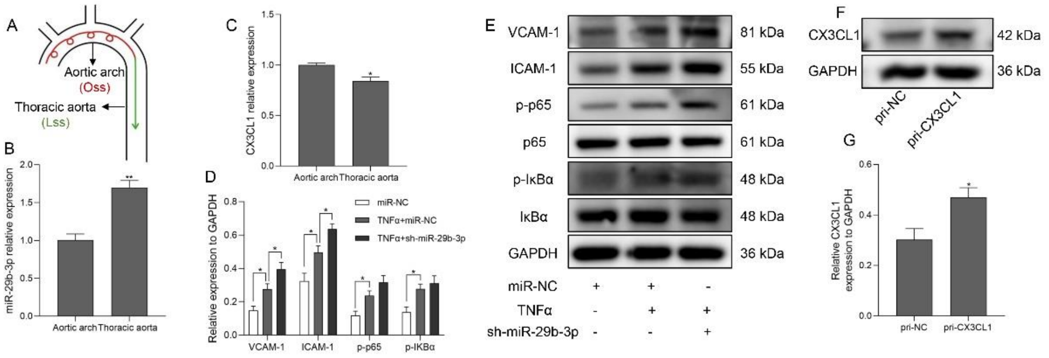

Fig. S3. (A) Schematic diagram of the aorta. The expression levels of miR-29b-3p (B) and CX3CL1 (C) in the aortic arch intima and thoracic aortic intima of ApoE$^{-/-}$ mice were determined by qRT-PCR. The protein (D and E) expression of VCAM-1, ICAM-1, p-p65, p65, p-IκBα and IκBα with/without miR-29b-3p knockdown in TNF-α-treated and non-treated HAECs. The protein (F and G) expression of CX3CL1 after HAECs were transfected with pri-CX3CL1 or pri-NC. The results are presented as mean ± SEM of three independent experiments. *$p < .05$, ** $p < .01$, *** $p < .001$.

Journal of Cell Science • Supplementary information

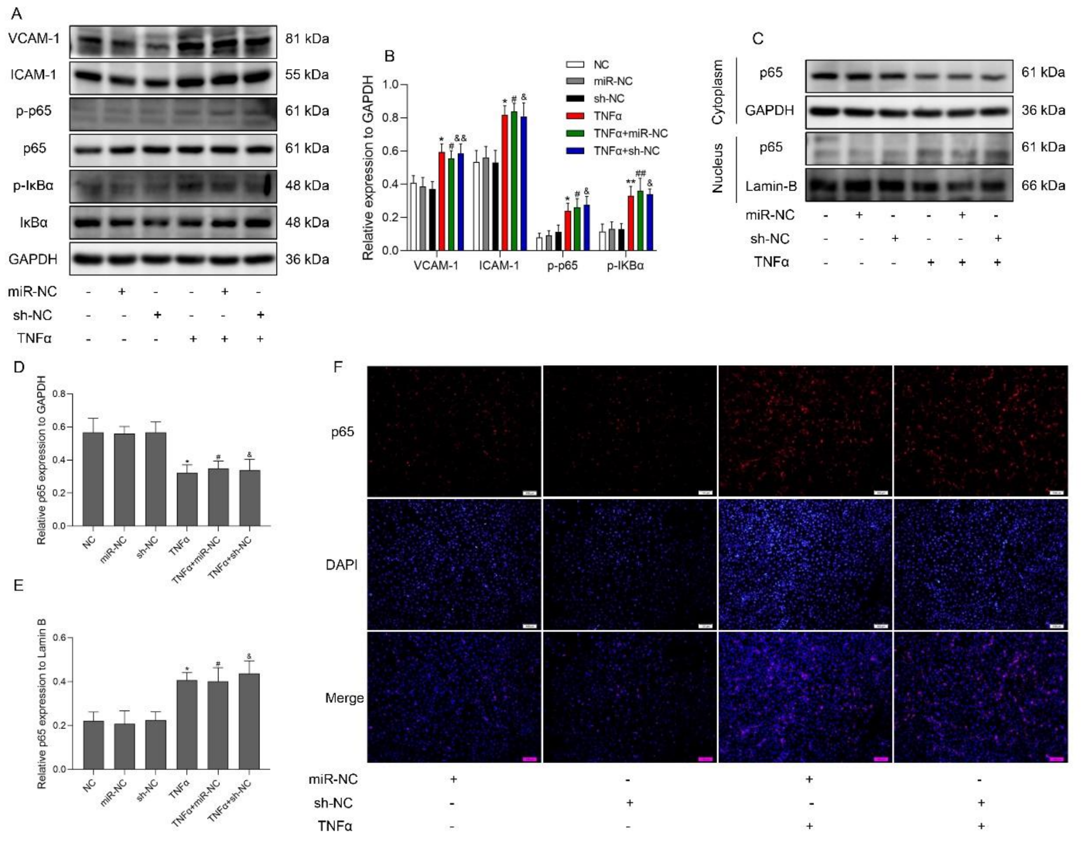

Fig. S4. HAECs were transfected with miR-NC or sh-NC in the presence or absence of TNF-α (10 ng/mL). (A and B) VCAM-1, ICAM-1, p-p65, p65, p-IκBα, and IκBα protein expression was determined by Western blotting. (C, D, and E) The protein expression of p65 in the cytoplasm and nucleus of HAECs. (F) The expression and localization of p65 in HAECs were determined by immunofluorescence. The results are presented as mean ± SEM of three independent experiments. $*p < .05$, $**p < .01$ vs. NC group; $^{\#}p < .05$, $^{\#\#}p < .01$ vs. m iR-NC group; $^{\&}p < .05$, $^{\&\&}p < .01$ vs. sh -NC group.

Journal of Cell Science • Supplementary information

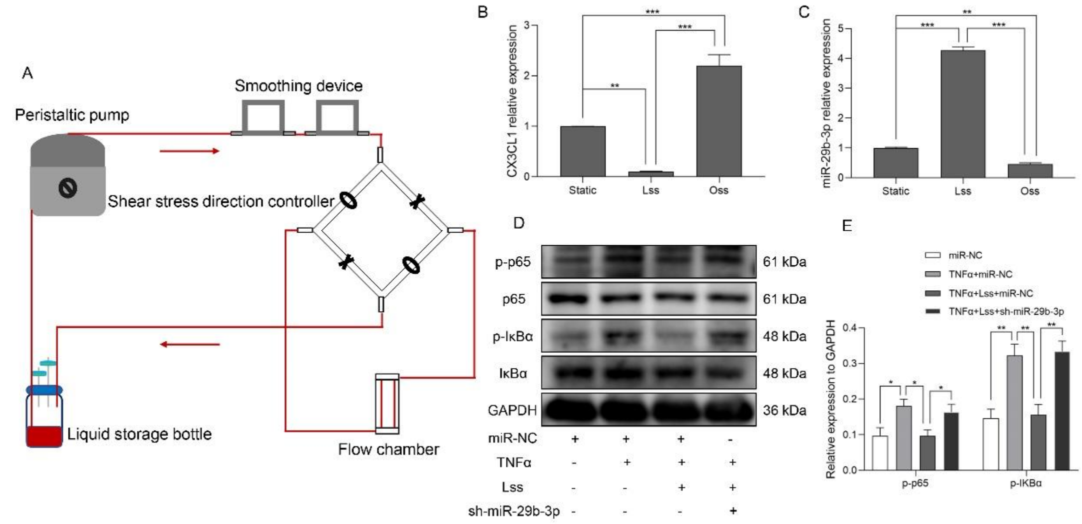

Fig. S5. (A) Schematic diagram of the instrument used to simulate Oss in vitro. The expression levels of CX3CL1 (B) and miR-29b-3p (C) in static, Lss-treated and Oss-treated HAECs were determined by qRT-PCR. HAECs were transfected with sh-miR-29b-3p or negative control in the presence or absence of TNF-α (10 ng/mL), and were then selectively subjected to Lss for 12 hours. (D and E) The protein expression of p-p65, p65, p-IκBα and IκBα were determined by Western blotting. The results are presented as mean ± SEM of three independent experiments. **p* < .05, ** *p* < .01, *** *p* < .001.

Journal of Cell Science • Supplementary information

Table S1. Down-regulated mRNAs in HAECs under LSS

Click here to download Table S1

Table S2. Down-regulated mRNAs in HUVECs under LSS

Click here to download Table S2

Table S3. ShRNAs sequences

Name	Sequnece (5’-3’)
sh-CX3CL1-1	CCGGCCCGGAGCTGTGGTAGTAATTCTCGAGAATTACTACCACAGCTCCGGGTTTTTG
sh-CX3CL1-2	CCGGCATCAAAGATACCTGTAGCTTCTCGAGAAGCTACAGGTATCTTTGATGTTTTTG
sh-CX3CL1-3	CCGGGCTGTGGTAGTAATTCATATGCTCGAGCATATGAATTACTACCACAGCTTTTTG
Negative control (sh-CX3CL1)	GTTCTCCGAACGTGTCACGTT
sh-miR-29b-3p	aacactgattgttatggtgctaCCGaacactgattgttatggtgctaTTTTT

Journal of Cell Science • Supplementary information

Table S4. Primer sequences

Gene Name	Forward Primer	Reverse Primer
CXCR4	TGTCATCTACACAGTCAACCTC	CAACATAGACCACCTTTTCAGC
APLN	CAGAGGGTCAAGGAATGGG	GAAAGGCATGGGTCCCTTAT
CX3CL1	TATCAACAGAACCAGGCATCAT	GACCACAGACTCGTCCATTC
CSF1	TGATTGACAGTCAGATGGAGAC	TAGCACACTGGATCTTTCAACT
HES1	AACACTGATTTTGGATGCTCTG	CACTGTCATTTCCAGAATGTCC
VCAM-1	CAGGCTGGAGATAGACTTACTG	CCTCAATGACAGGAGTAAAGGT
ICAM-1	TGCAAGAAGATAGCCAACCAAT	GTACACGGTGAGGAAGGTTTTA
β-actin	GGCCAACCGCGAGAAGATGAC	GGATAGCACAGCCTGGATAGCAAC
hsa-miR-29b-3p	GCCGCGCGTAGCACCATTTGAAATCAG	
hsa-miR-29a-3p	CGCGCTAGCACCATCTGAAATCGGT	
hsa-miR-29c-3p	CGCGCGTAGCACCATTTGAAATCGGT	
hsa-miR-497-5p	CCGCAGCAGCACACTGTGGTTTGT	
hsa-miR-424-5p	GGCGCGCAGCAGCAATTCATGTTTTGA	
U6	CGCAGAGAAGATTAGCATGGCCCCTG	

Journal of Cell Science • Supplementary information

Figure S6. Blot Transparency.

Figure 1

CX3CL1

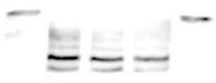

GAPDH

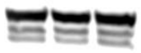

Journal of Cell Science • Supplementary information

Figure 2

C-CX3CL1

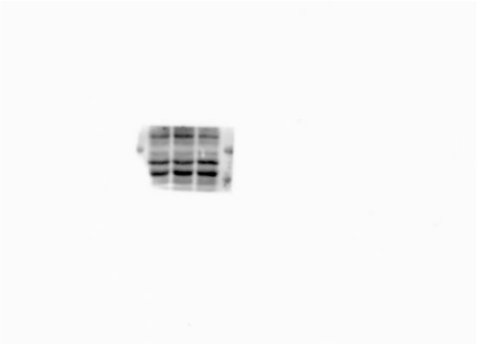

C-GAPDH

C-ICAM-1

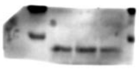

C-VCAM-1

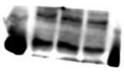

Journal of Cell Science • Supplementary information

I-CX3CL1

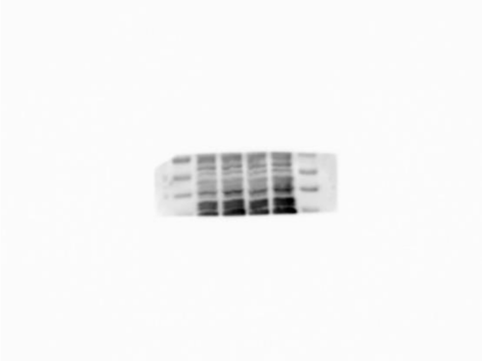

I-GAPDH

I-ICAM-1

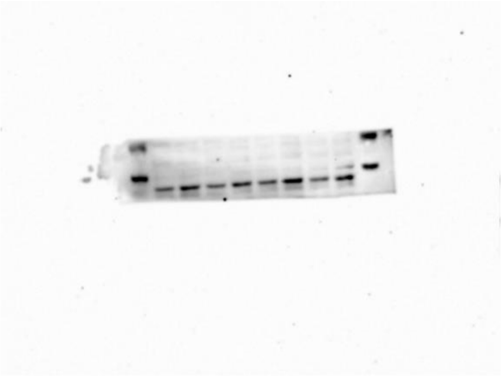

I-VCAM-1

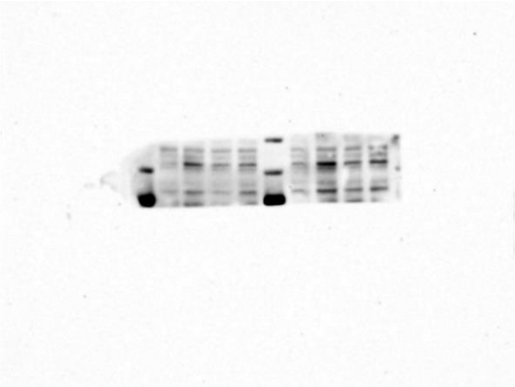

Journal of Cell Science • Supplementary information

Figure 3

G-CX3CL1-left

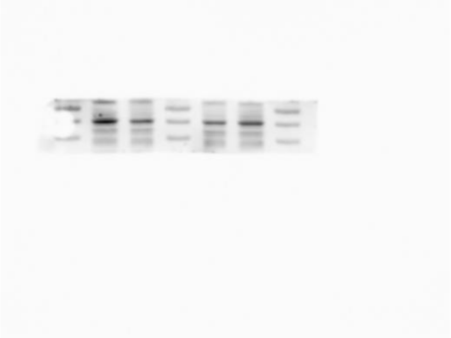

G-GAPDH-left

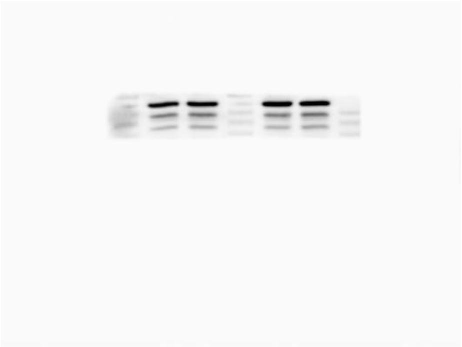

K-CX3CL1-right

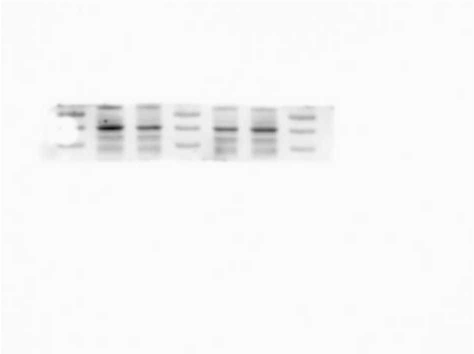

K-GAPDH-right

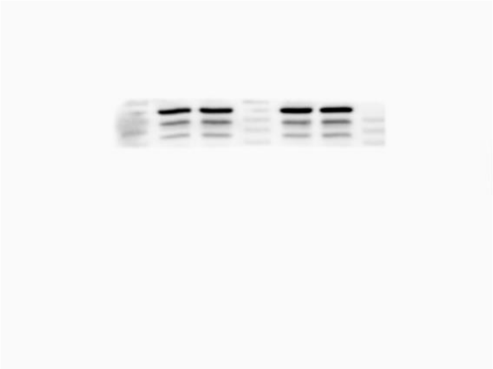

Journal of Cell Science • Supplementary information

Figure 4

C-CX3CL1

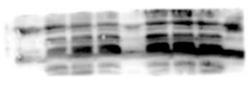

C-GAPDH

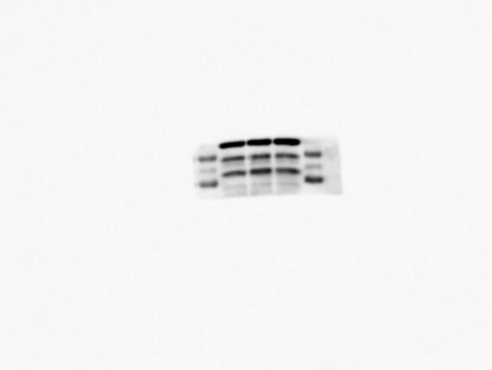

F-GAPDH

F-ICAM-1

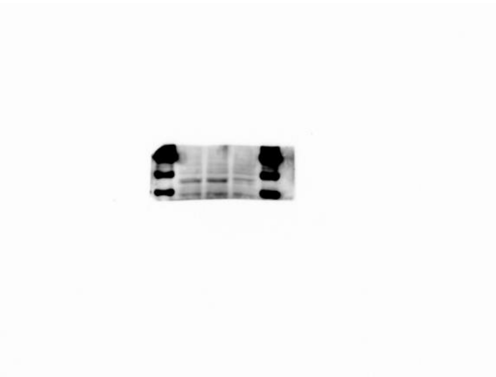

Journal of Cell Science • Supplementary information

F-VCAM-1

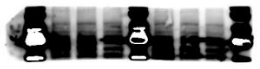

K-CX3CL1

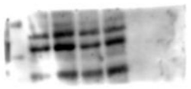

K-GAPDH

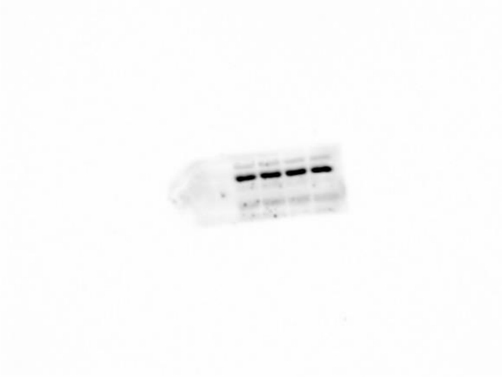

K-ICAM-1

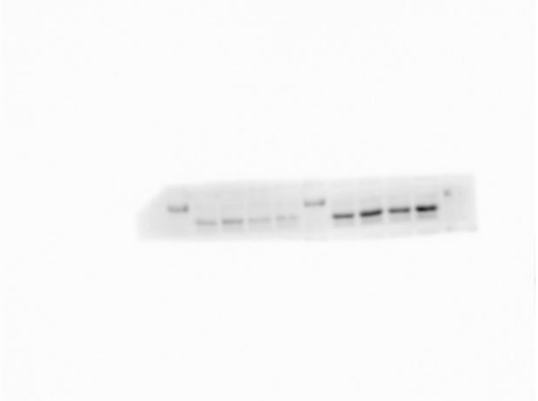

Journal of Cell Science • Supplementary information

K-VCAM-1

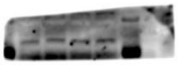

P-CX3CL1

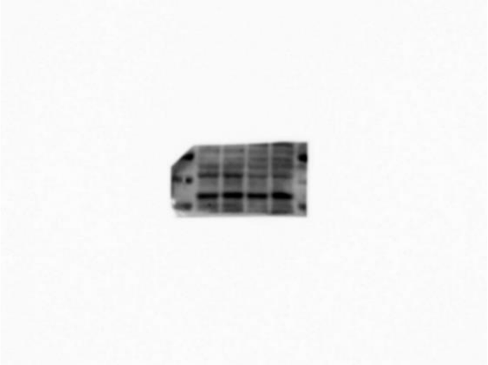

P-GAPDH

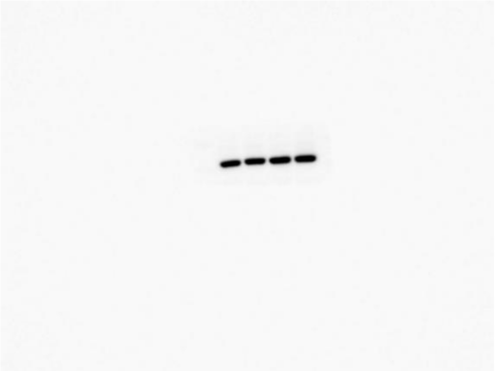

P-ICAM-1

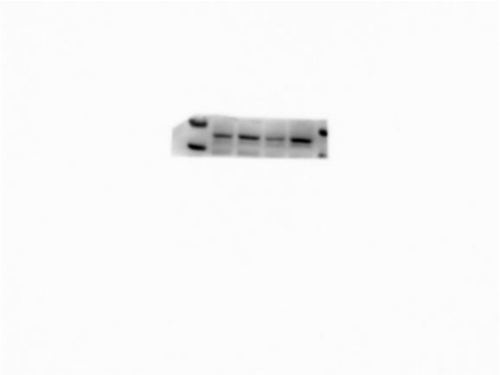

Journal of Cell Science • Supplementary information

P-VCAM-1

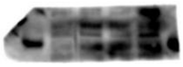

Journal of Cell Science • Supplementary information

Figure 5

A-GAPDH

A-ICAM-1

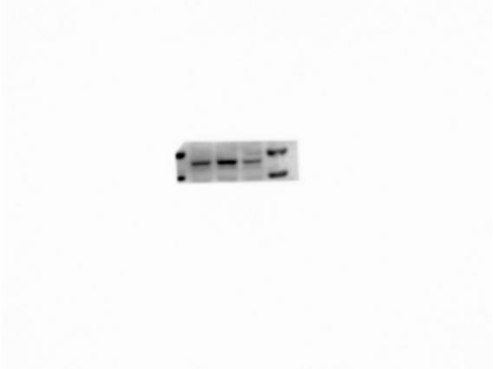

A-IκBα

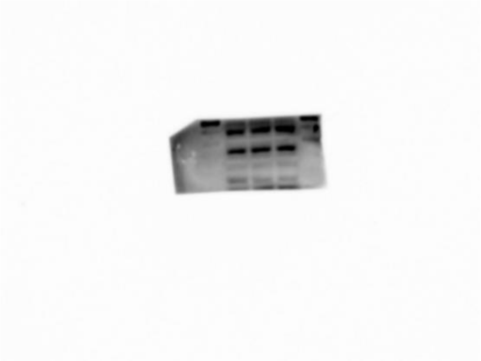

A-p65

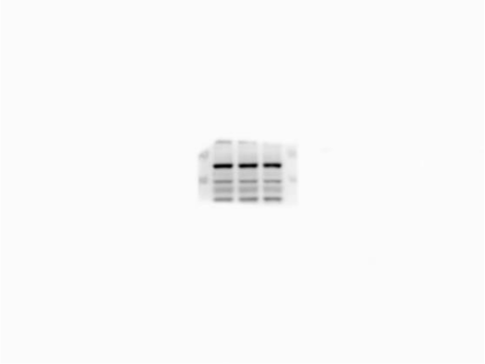

Journal of Cell Science • Supplementary information

A-p-IκBα

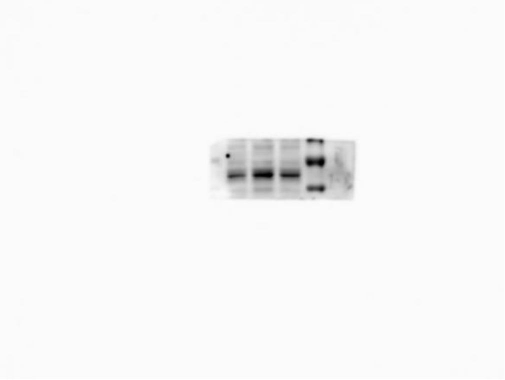

A-p-p65

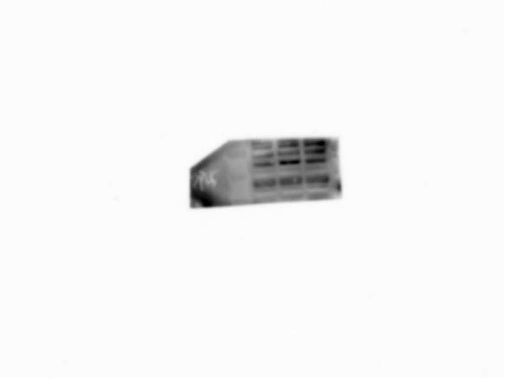

A-VCAM-1

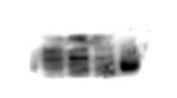

C-GAPDH

Journal of Cell Science • Supplementary information

C-LaminB

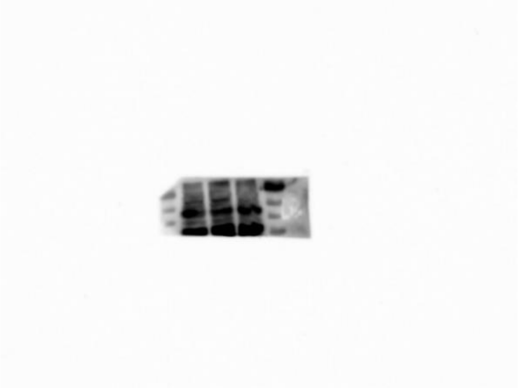

C-p65(Cytoplasm)

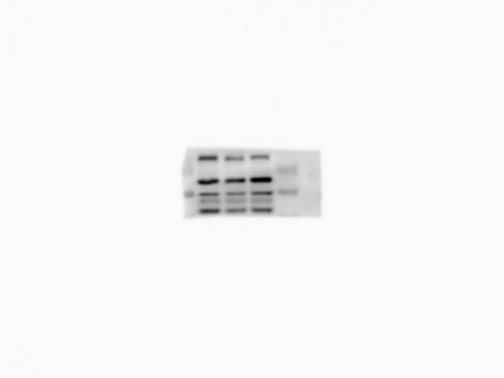

C-p65(Nucleus)

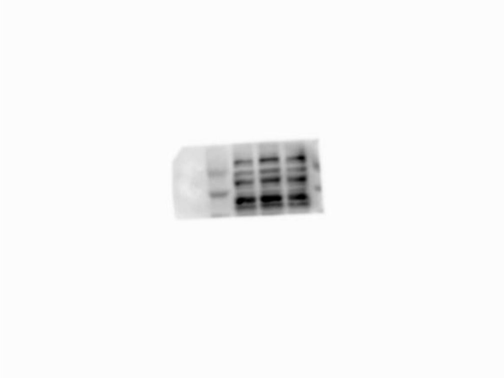

G-GAPDH-left

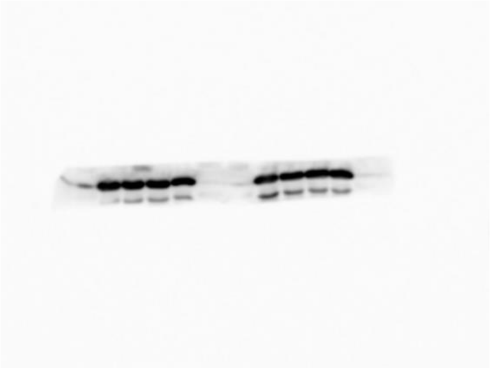

G-ICAM-1

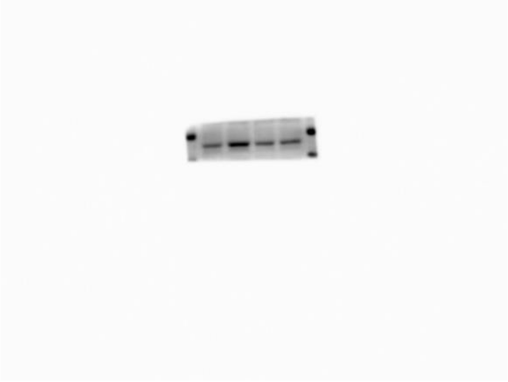

G-IκBα

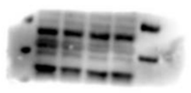

G-p65-left

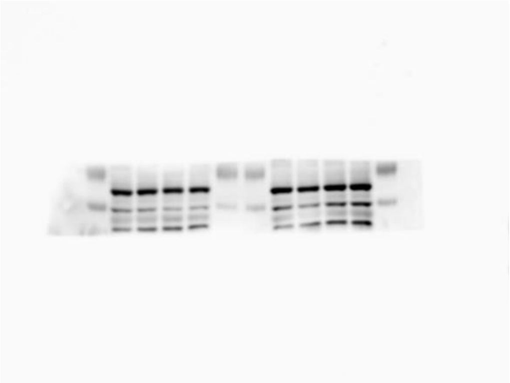

G-p-IκBα

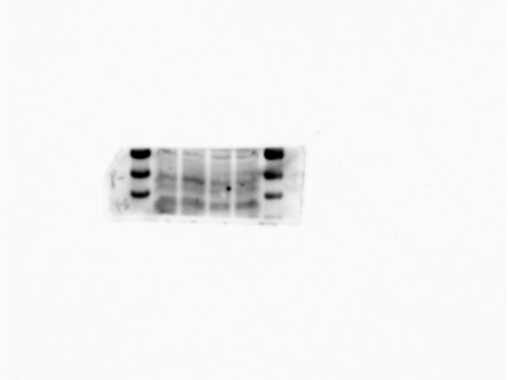

G-p-p65

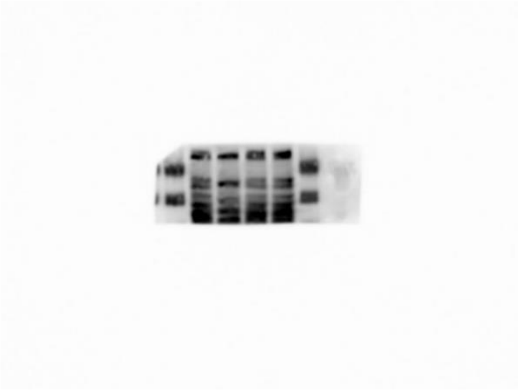

G-VCAM-1

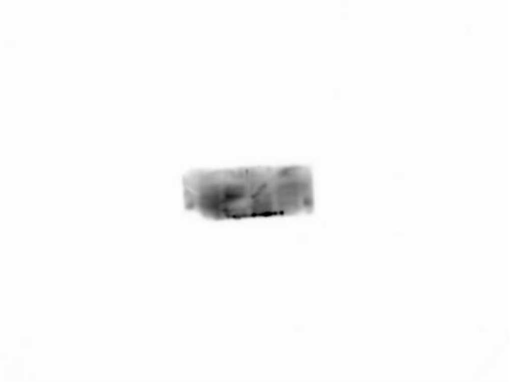

I-GAPDH-right

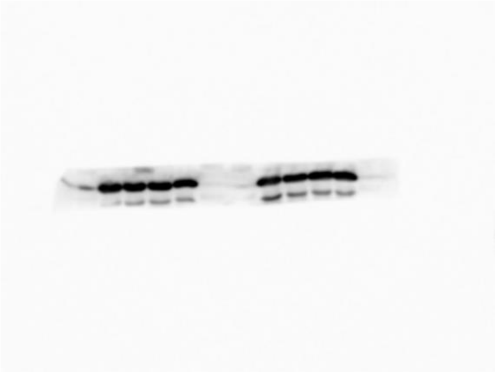

I-LaminB-right

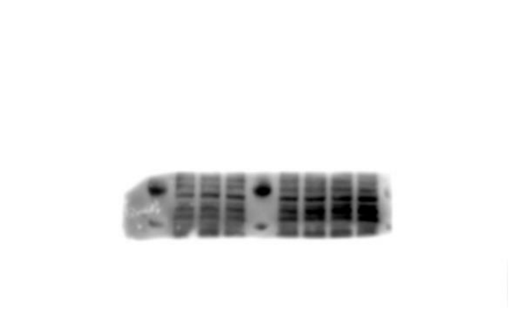

Journal of Cell Science • Supplementary information

I-p65(Cytoplasm)-right

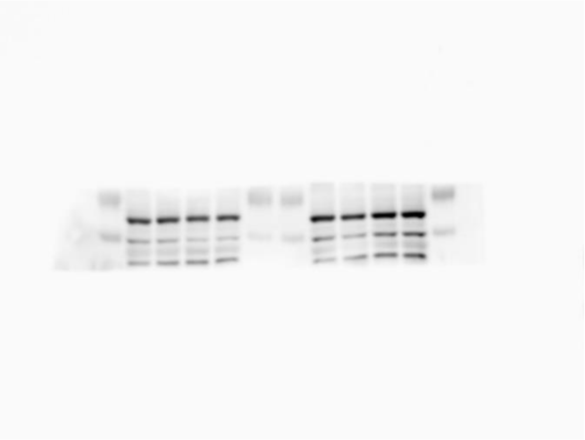

I-p65(Nucleus)

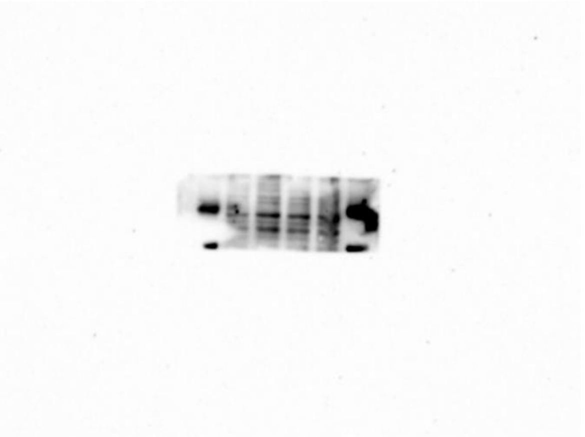

Journal of Cell Science • Supplementary information

Figure 6

J-CX3CL1

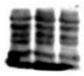

J-GAPDH

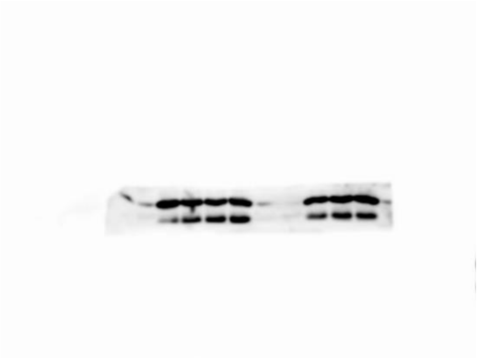

J-ICAM-1

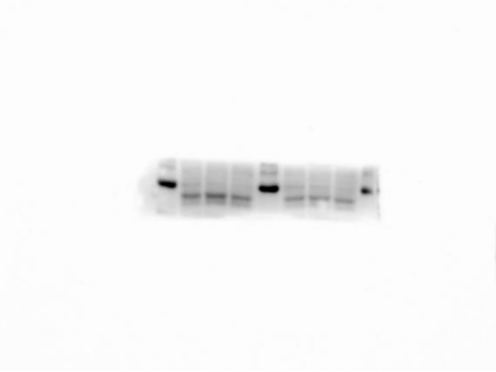

J-VCAM-1

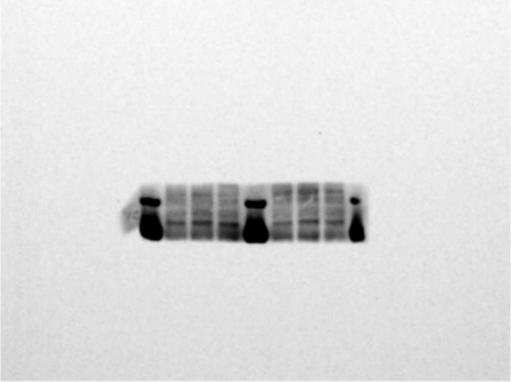

Figure S2

C-VCAM-1

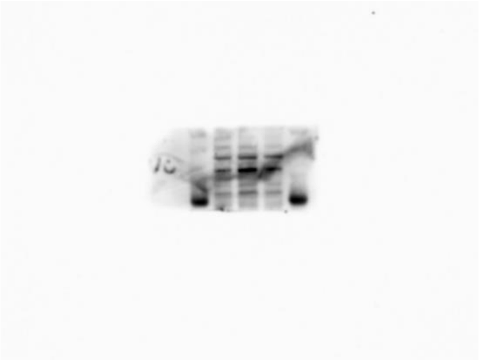

C-ICAM-1

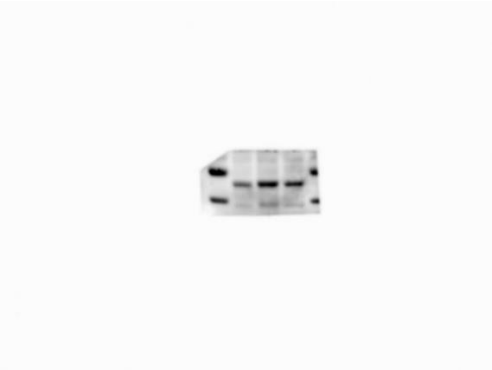

C-p-p65

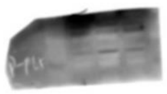

C-p65

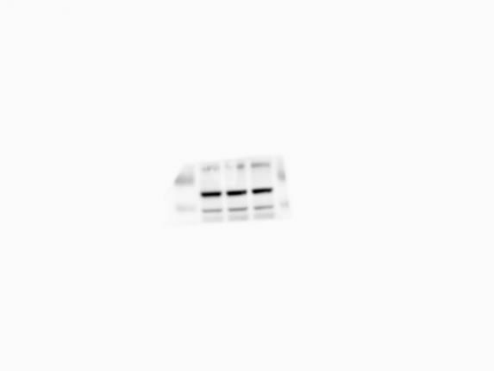

C-p-IκBα

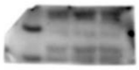

C-IκBα

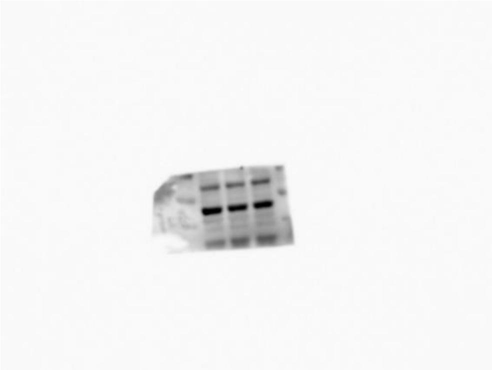

C-GAPDH

D- p-p65

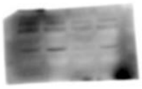

Journal of Cell Science • Supplementary information

D-p65

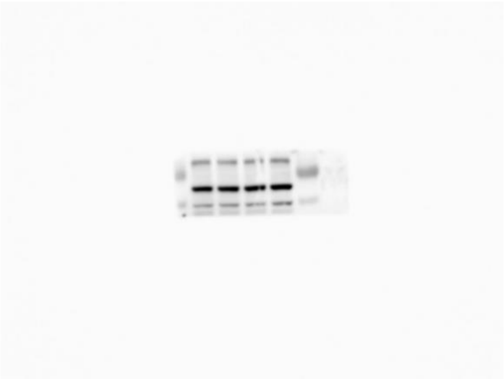

D- p-IκBα

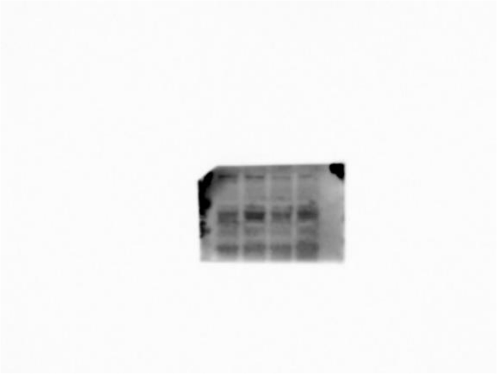

D- IκBα

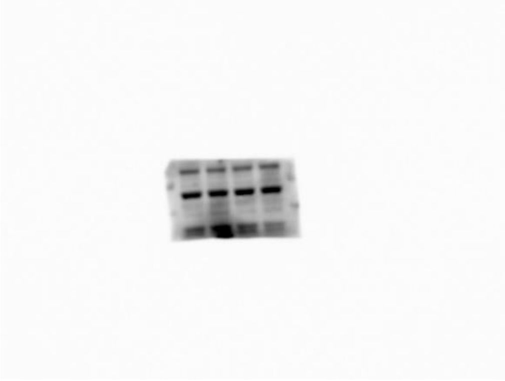

D- GAPDH

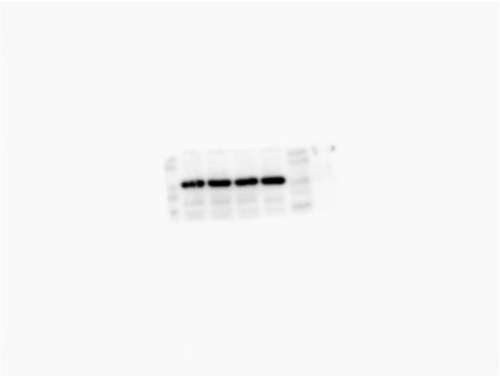

G-CX3CL1

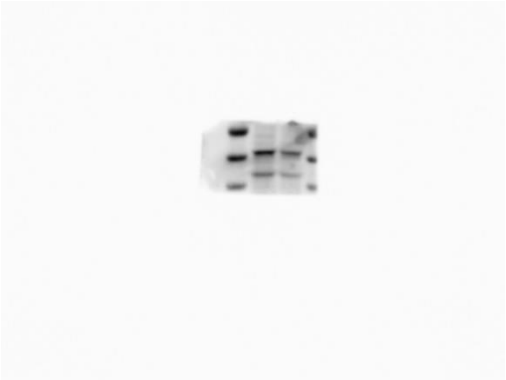

G-GAPDH

Figure S3

E-VCAM-1

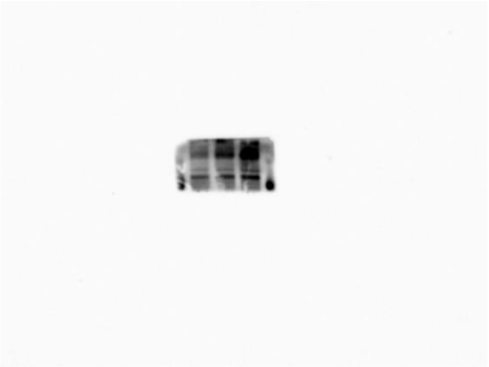

E-ICAM-1

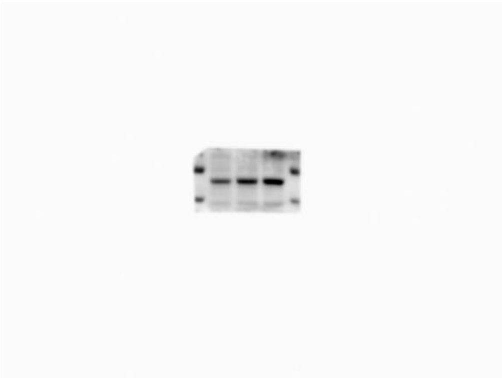

E-p-p65

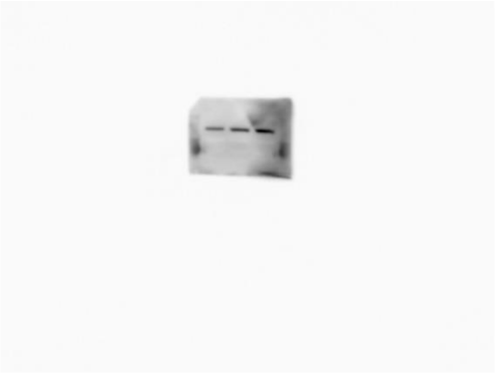

E-p65

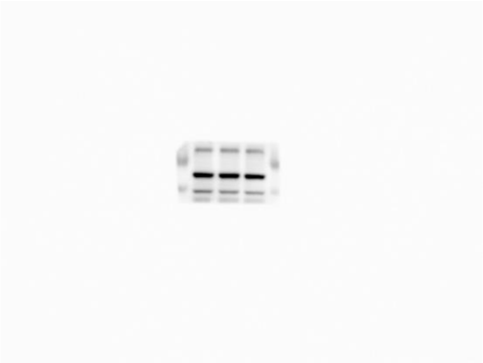

E-p- IκBα

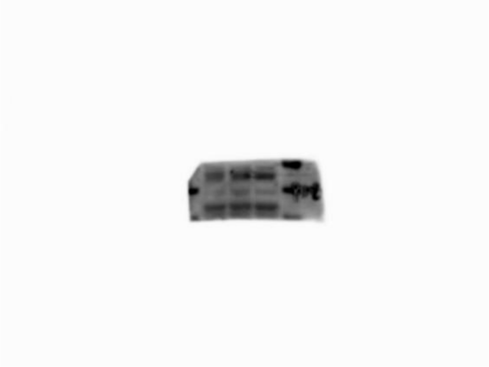

E- IκBα

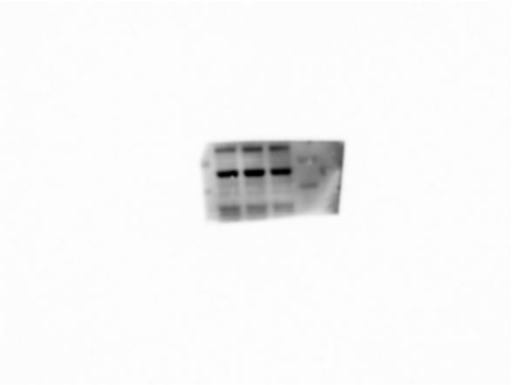

E-GAPDH

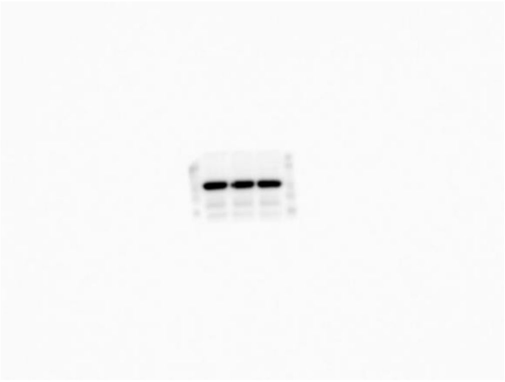

F-CX3CL1

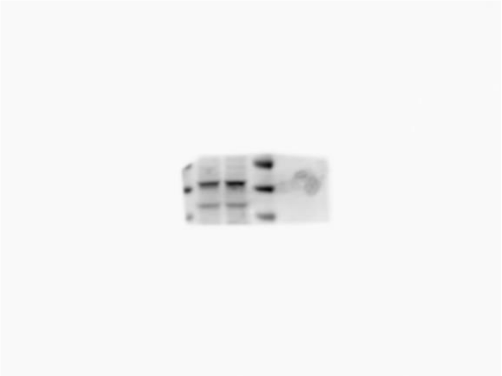

Journal of Cell Science • Supplementary information

F-GAPDH

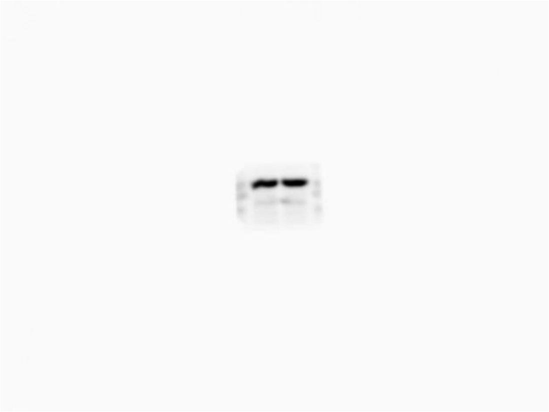

Journal of Cell Science • Supplementary information

Figure S4

A-VCAM-1

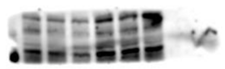

A-ICAM-1

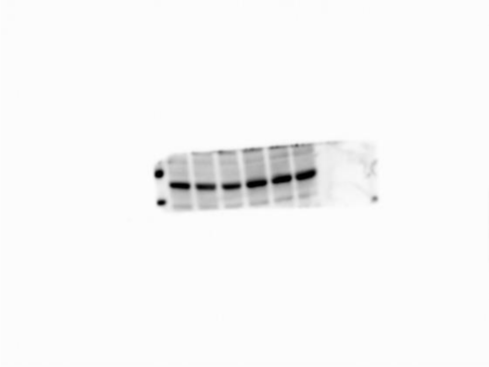

A-p-p65

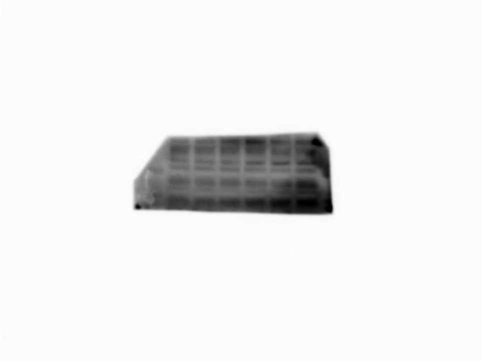

A-p65

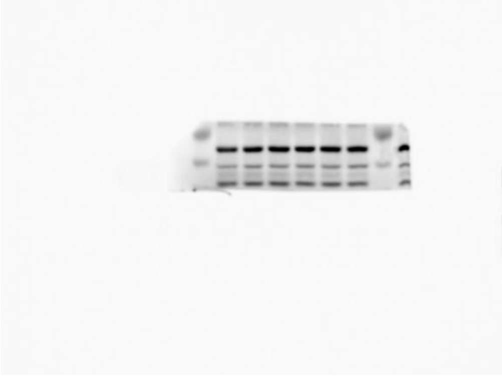

A-p-IκBα

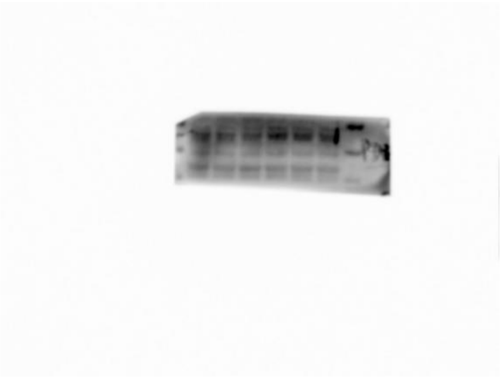

A-IκBα

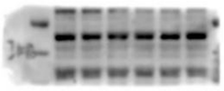

A-GAPDH

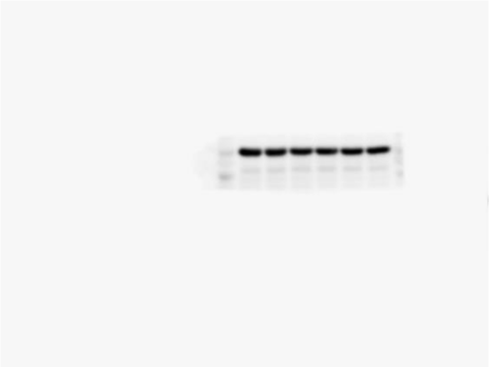

C-p65 (Cytoplasm)

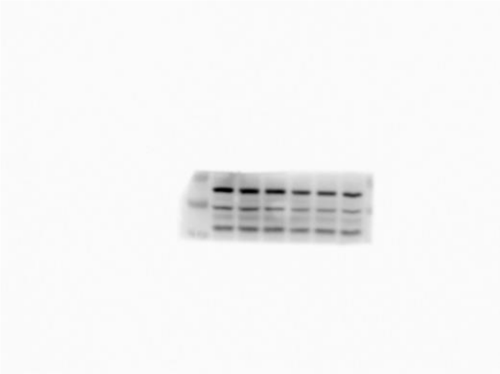

C-GAPDH

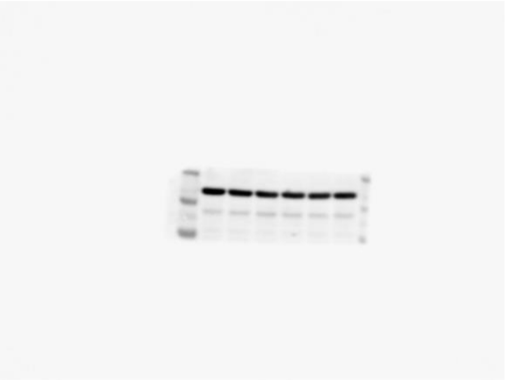

C-P65 (Nucleus)

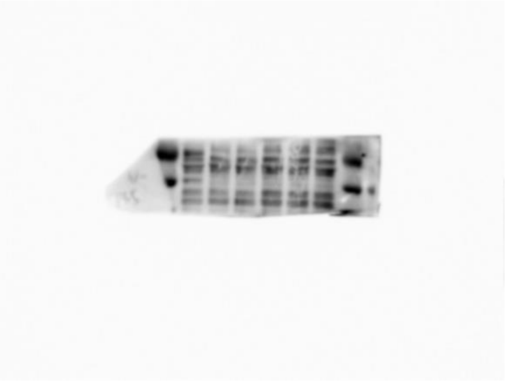

C-Lamin-B

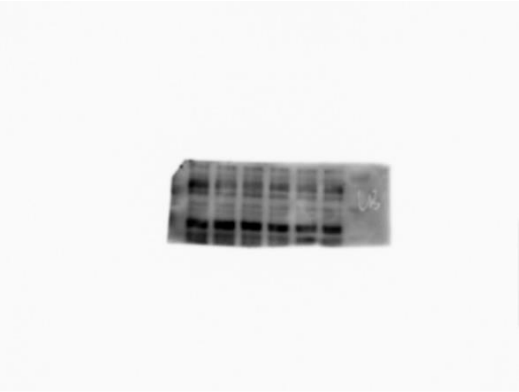

Figure S5

D-p-p65-right

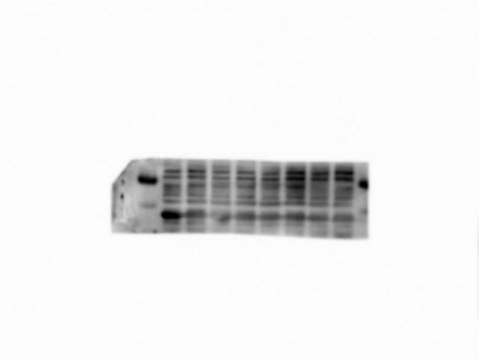

D-p65

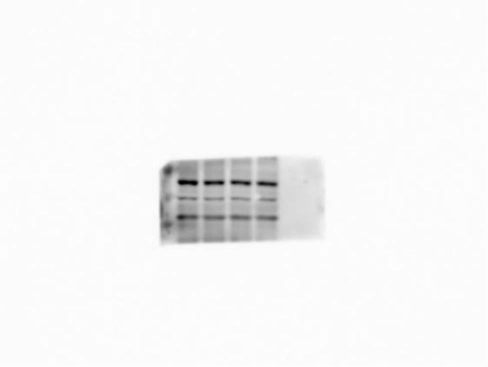

D-p-IκBα

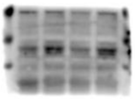

D-IκBα

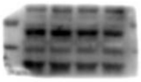

Journal of Cell Science • Supplementary information

D-GAPDH

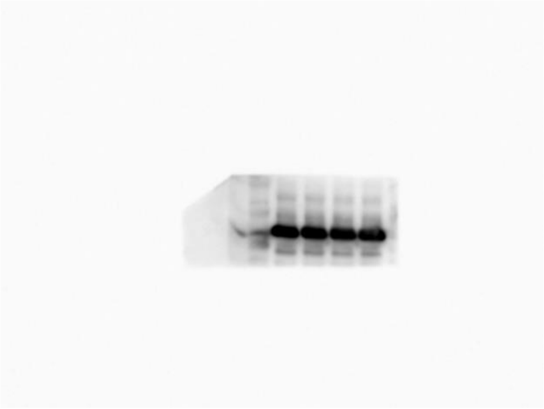

Journal of Cell Science • Supplementary information
