# Supplementary material for: Laminar shear stress alleviates monocyte adhesion and atherosclerosis development via miR-29b-3p/CX3CL1 axis regulation
Source: J Cell Sci. 2022 Jul 22;135(14):jcs259696. doi: 10.1242/jcs.259696 (PMC9450891; doi:10.1242/jcs.259696)
Supplement: Reviewer comments [file joces-135-259696_review_history.pdf]

Laminar shear stress alleviates monocyte adhesion and atherosclerosis development via miR-29b-3p/CX3CL1 axis regulation

Luya Pu, Qingyu Meng, Shuai Li, Yaru Wang, Banghao Sun, Bin Liu and Fan Li
DOI: 10.1242/jcs.259696

Editor: Michael Way

Review timeline

Original submission:	18 December 2021
Editorial decision:	8 February 2022
First revision received:	18 March 2022
Editorial decision:	19 May 2022
Second revision received:	15 June 2022
Accepted:	17 June 2022

Original submission

First decision letter

MS ID#: JOCES/2021/259696

MS TITLE: Laminar shear stress alleviates monocyte adhesion and atherosclerosis development via miR-29b-3p/CX3CL1 axis regulation

AUTHORS: Luya Pu, Qingyu Meng, Shuai Li, Yaru Wang, Banghao Sun, Bin Liu, and Fan Li
ARTICLE TYPE: Research Article

We have now reached a decision on the above manuscript.

To see the reviewers' reports and a copy of this decision letter, please go to: https://submit-jcs.biologists.org and click on the 'Manuscripts with Decisions' queue in the Author Area. (Corresponding author only has access to reviews.)

As you will see, the reviewers raise a number of substantial criticisms that prevent me from accepting the paper at this stage. They suggest, however, that a revised version might prove acceptable, if you can address their concerns. If you think that you can deal satisfactorily with the criticisms on revision, I would be pleased to see a revised manuscript. We would then return it to the reviewers.

We are aware that you may be experiencing disruption to the normal running of your lab that makes experimental revisions challenging. If it would be helpful, we encourage you to contact us to discuss your revision in greater detail. Please send us a point-by-point response indicating where you are able to address concerns raised (either experimentally or by changes to the text) and where you will not be able to do so within the normal timeframe of a revision. We will then provide further guidance. Please also note that we are happy to extend revision timeframes as necessary.

Please ensure that you clearly highlight all changes made in the revised manuscript. Please avoid using 'Tracked changes' in Word files as these are lost in PDF conversion.

© 2022. Published by The Company of Biologists under the terms of the Creative Commons Attribution License (https://creativecommons.org/licenses/by/4.0/).

I should be grateful if you would also provide a point-by-point response detailing how you have dealt with the points raised by the reviewers in the 'Response to Reviewers' box. Please attend to all of the reviewers' comments. If you do not agree with any of their criticisms or suggestions please explain clearly why this is so.

Reviewer 1

Advance summary and potential significance to field

This is an interesting study addressing previous work that have demonstrated flow induced regulation of the chemokine CX3CL1 in endothelial cells and its implication in vascular inflammation. The authors significantly extend the current knowledge by presenting an miRNA mediated molecular mechanism of CX3CL1 downregulation and by providing evidence for the protective role of this mechanism in an in vivo model.

Some issues remain to be addressed either experimentally or by discussion.

Comments for the author

Major
1) CX3CL1 appeared as 43 kDa molecule. According to most other studies the size of full length CX3CL1 is about 95 kDa while cleaved CX3CL1 migrates at 80 KDa. It is possible that the authors have investigated another cleavage product.
For this reason, the whole Western bots must be shown exemplary in the first figure and if necessary due to the presence of additional bands also in the supplements for other blots.
2) There seem to be CX3CL1 dependent effects on ICAM-1, VCAM-1 and NFKappaB. This raises the question whether CX3CR1 signaling is involved. Do the authors assume an autocrine amplification loop via the CX3CL1/CX3CR1 axis? What happens if exogenous CX3CL1 is added or when the CX3CL1/CX3CR1 axis is inhibited by an antibody or a small molecule? Is there also an effect on VCAM-1 and ICAM-1 expression or NFKappaB activation?
3) Can the authors exclude a direct CX3CL1 independent effect of the miRNA on NFKappaB activation?
4) Was the agomir miRNA tested for CX3CL1 downregulation in vitro?
5) Fig 6 shows that the miRNA is downregulated in the aortic tissue. It is not clear whether this downregulation is related to alterations in shear stress.
It could also be that other cells not exposed to flow downregulate miRNA. Was there difference in regions of laminar or turbulent or low flow.
6) CX3CL1 is also present on smooth muscle cells in the inflamed lesions.
It could well be that also here the agomir miRNA may alleviate CX3CL1 expression and its inflammatory signaling.
7) An antagomir miRNA could have been tested to block the protective effects of endogenous miR29b-3p in vivo. There still remains some expression of miR29b-3p even after HFD and further downregulation my exacerbate the lesion formation.
8) The n numbers must be specified for each experiment, especially when SEM is used instead of SD.
9) It should be stated that each shown western blot result is representative for at least three independent experiments.

Minor
1) The font size in the Figures is sometimes very small. All legends should be clearly readable
2) Second page of results was the CX3CL1 expression “restored” after sh-CX3CL1 transfection? This should be better explained.
3) Page numbers are missing and would have made the review easier.

Reviewer 2

Advance summary and potential significance to field

In the study by Li and co-workers, the authors examined a mechanism by which laminar shear stress (LSS) modulates adhesion molecule expression and monocyte adhesion in endothelial cells.

© 2022. Published by The Company of Biologists under the terms of the Creative Commons Attribution License (https://creativecommons.org/licenses/by/4.0/).

Specifically, they examined whether miR-29b-3p, an miRNA that has increased expression in endothelial cells subjected to LSS, suppresses expression of VCAM-1 and ICAM-1 through targeting of CX3CL1. The authors show that expression of CX3CL1, VCAM-1, and ICAM-1 was decreased in cultured endothelial cells subjected to LSS.
Subsequently, they show that short hairpin RNA against CX3CL1 suppressed TNFalpha-induced expression of VCAM-1 and ICAM-1 and monocyte adhesion to endothelial cells. In silico analysis revealed that miR-29b-3p could potentially target CX3CL1 3'UTR, which was verified in luciferase reporter assays of wild type and mutant CX3CL1 3'UTRs. Enhanced expression of miR-29b in cultured endothelial cells decreased TNFalpha-induced expression of CX3CL1 and downstream mediators of inflammation VCAM-1 and ICAM-1. The authors provide evidence that the mechanism by which LSS and miR-29b suppressed monocyte adhesion to cultured endothelial cells involved inhibition of NF-kappaB signaling. Finally, hypercholesterolemic mice that were injected with agomiR-29b via tail vein had reduced aortic expression of CX3CL1, VCAM-1, and ICAM-1 and less aortic atherosclerotic plaques compared to mice injected with negative control agomiR.
The study provides solid evidence for a novel role of the miR-29b/CX3CL1 pathway in LSS-mediated changes in endothelial phenotype vitro. However, it would be helpful if the authors could provide more data on the negative control experiments for the in vitro studies. For the in vivo studies, a stronger case for the physiologic relevance of the miR-29b/CX3CL1 mechanism could be made with data demonstrating that miR-29b/CX3CL1 expression differed in endothelial cells from aortic segments exposed to LSS compared to those exposed to disturbed flow. The authors should also provide more data showing exogenous miR-29b suppressed CX3CL1 expression in aortic endothelial cells versus whole aortic tissues. Furthermore, the effect of exogenous miR-29b on lipid levels suggests that the mechanism responsible for the anti-atherosclerotic effect of agomir-miR-29b may not involve the miR-29b/CX3CL1 pathway. Finally, it is not clear how the current study can be reconciled with a study from 2014 published in the International Journal of Cardiology (Zhu et al., vol. 176, pages 764-770), which showed that ApoE-/- mice fed a high fat diet had increased aortic expression of miR-29b, and miR-29b mimic enhanced ICAM-1 and NFkappaB expression in cultured endothelial cells.

Comments for the author

Specific comments/questions:
1. It is surprising that the authors did not discuss or reference any of the studies of LSS and KLF2; these studies have identified several LSS-responsive miRNAs involved in modulating endothelial cell phenotype.
2. Based on their in vivo studies, it is not clear that the authors can conclude that LSS inhibits AS progression by regulating CX3CL1. The in vivo experiments do not show association between LSS, miR-29b, and CXCL1 in mouse aortic endothelial cells. In addition, exogenous miR-29b altered lipid levels of the ApoE-/- mice on HFD, so it is not clear that the miR-29b/CX3CL1 pathway is relevant in this model
3. Figure 1: it is very difficult to read words in panels A-J, F-G, even after magnification. Please indicate amount LSS to which the cells were exposed and rationale for this amount. In panel E, it is not clear why the number of downregulated genes went from 155 to 130 in HAECs and 853 to 828 in HUVECs.
4. Figure 2 shows gene expression and monocyte adhesion in cells with only one negative control. What about the other negative controls for these experiments? For example, data for gene expression in cells treated with TNFalpha plus sh-NC, cells treated with TNFalpha plus LSS and pri-NC should be shown. Please provide western blot data for panel G.
5. Figure 3 – please clarify what was used as negative control mimic for luciferase reporter gene experiment.
Please clarify why only the low concentration of sh-miR-29b suppressed miR-29b expression.
6. Figure 4 – similar comment about negative control data for pri-miR-NC as stated in #4 above. What about data for TNFalpha-pri-miR-NC or TNFalpha plus LSS and miR-NC? It is not clear that the quantitative data for VCAM-1 in panel G reflect blot in panel F.
7. Figure 5 – it is hard to see from the immunofluorescence images provided in panel F that LSS decreased TNFalpha-induced nuclear translocation of p65. Can clearer or more magnified images be provided? For experiments depicted in panels G-L, it would be helpful to see all of the data for negative control conditions e.g. TNFalpha plus NC. Also, it would be helpful to see data on TNFalpha plus sh-miR-29b.

© 2022. Published by The Company of Biologists under the terms of the Creative Commons Attribution License (https://creativecommons.org/licenses/by/4.0/).

8. Figure 6 – please clarify whether the sequences for mouse and human miR-29b are homologous and whether mouse and human CX3CL1 are homologous. Please provide data for mice injected with negative control agomiR. Since in vitro studies showed that miR-29b suppressed monocyte adhesion to endothelial cells, can the authors provide information on monocyte infiltration into vessel wall in agomiR-treated mice?
Please clarify why lipid levels are altered by exogenous miR-29b and how this might impact findings. Please provide additional information on how plaque area and lipid deposition area was quantified in whole aortas of mice. Since the analysis of gene expression involved whole aorta tissues, it is not clear whether miR-29b reduced endothelial cell inflammation in vivo.
9. In the Discussion section, first paragraph, the authors state that the pharmacologic mechanism of statins in treatment of AS is to improve the effect of turbulence on endothelial cell function by inhibiting activity of YAP/TAZ. This statement is not accurate since it is well recognized that the primary pharmacologic mechanism of statins in treatment of AS is lipid lowering.

First revision

Author response to reviewers' comments

#Reviewer 1 Advance Summary and Potential Significance to Field:
This is an interesting study addressing previous work that have demonstrated flow induced regulation of the chemokine CX3CL1 in endothelial cells and its implication in vascular inflammation. The authors significantly extend the current knowledge by presenting an miRNA mediated molecular mechanism of CX3CL1 downregulation and by providing evidence for the protective role of this mechanism in an in vivo model.

Some issues remain to be addressed either experimentally or by discussion.

Reviewer 1 Comments for the Author:
Major
1) CX3CL1 appeared as 43 kDa molecule. According to most other studies the size of full length CX3CL1 is about 95 kDa while cleaved CX3CL1 migrates at 80 KDa. It is possible that the authors have investigated another cleavage product. For this reason, the whole Western bots must be shown exemplary in the first figure and if necessary due to the presence of additional bands also in the supplements for other blots.

Thank you very much for your professional comment. Based on your suggestion, the whole western bolt have been shown below. In addition, after reviewing the references, we found that the western blot of CX3CL1 provided by many studies on membrane- bound CX3CL1 were located to 42 kDa (PMID: 30237497, PMID: 32664984, PMID: 30607245 and PMID: 29311834).

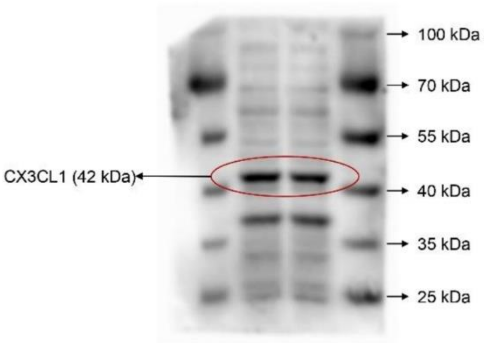

2) There seem to be CX3CL1 dependent effects on ICAM-1, VCAM-1 and NFKappaB. This raises the question whether CX3CR1 signaling is involved. Do the authors assume an autocrine amplification loop via the CX3CL1/CX3CR1 axis? What happens if exogenous CX3CL1 is added or when the CX3CL1/CX3CR1 axis is inhibited by an antibody or a small molecule? Is there also an effect on VCAM-1 and ICAM-1 expression or NFKappaB activation?

© 2022. Published by The Company of Biologists under the terms of the Creative Commons Attribution License (https://creativecommons.org/licenses/by/4.0/).

Thank you very much for your professional comment. Based on your suggestion, CX3CR1 expression in HAECs was inhibited by transfection of small interfering RNA (si-CX3CR1). As shown in **Figures S2A, B and C**, decreased expression of CX3CR1 significantly inhibited TNFα-stimulated upregulation of VCAM-1 and ICAM-1 expression, as well as the activation of NF-κB signaling pathway.

3) Can the authors exclude a direct CX3CL1 independent effect of the miRNA on NFKappaB activation?

Thank you very much for your professional comment. To exclude a direct CX3CL1 independent effect of the miRNA on NFKappaB activation, pri-miR-29b-3p and pri- CX3CL1 were co-transfected in TNFα-activated HAECs. As shown in **Figures S2D and E**, in TNFα-activated HAECs, overexpression of CX3CL1 abrogated the inhibitory effect of miR-29b-3p on NF-κB signaling pathway.

4) Was the agomir miRNA tested for CX3CL1 downregulation in vitro?

Thank you very much for your professional comment. As shown in **Figures S2F, G and H**, agomir-miRNA-29b-3p could inhibit the mRNA and protein expression levels of CX3CL1 in vitro.

5) Fig 6 shows that the miRNA is downregulated in the aortic tissue. It is not clear whether this downregulation is related to alterations in shear stress. It could also be that other cells not exposed to flow downregulate miRNA. Was there difference in regions of laminar or turbulent or low flow.

Thank you very much for your professional comment. Both RNAs and proteins from aortic tissue used in this study were extracted from the aortic intima. And the main components of the aortic intima are endothelial cells. In order to link Lss with miR- 29b-3p /CX3CL1 in vivo, this study investigated the expression levels of miR-29b-3p and CX3CL1 in the intima of the aortic arch of $ApoE^{-/-}$ mice exposed to turbulence (Oss) and the intima of the descending thoracic aorta exposed to Lss. As shown in **Figures S3A, B and C**, the expression of miR-29b-3p in the thoracic aortic intima exposed to Lss was up-regulated, whereas the expression of CX3CL1 was down- regulated compared with the aortic arch. This is consistent with in vitro results.

6) CX3CL1 is also present on smooth muscle cells in the inflamed lesions. It could well be that also here the agomir miRNA may alleviate CX3CL1 expression and its inflammatory signaling.

Thank you very much for your professional comment. Following your suggestion, we examined the effect of agomir-miRNA-29b-3p on CX3CL1 expression in human aortic smooth muscle cells. The human aortic smooth muscle cell line was obtained from the Department of Epidemiology and Biostatistics, Jilin University. As shown below, the mRNA expression level of CX3CL1 was up-regulated after agomir-miRNA-29b-3p (100 nM, 24h) transfection into human smooth muscle cells.

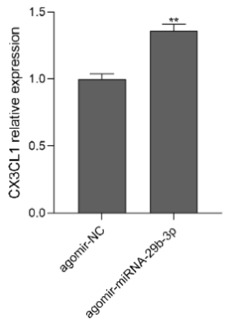

7) An antagomir miRNA could have been tested to block the protective effects of endogenous miR29b-3p in vivo. There still remains some expression of miR29b-3p even after HFD and further downregulation my exacerbate the lesion formation.

Thank you very much for your professional comment. Your suggestion is very meaningful to improve the quality of the article. But I am very sorry that the experimental period of animal experiments is too long (at least 5 months). Due to the graduation time requirement stipulated by the school, we do not have enough time to complete the animal experiment you suggested. However, we

© 2022. Published by The Company of Biologists under the terms of the Creative Commons Attribution License (https://creativecommons.org/licenses/by/4.0/).

explored the effect of sh- miR-29b-3p on the inflammatory response of TNFα-stimulated HAECs in vitro. As shown in **Figures S3D and E**, the decreased expression of miR-29b-3p significantly aggravated the TNFα-induced upregulation of the protein expression levels of VCAM- 1 and ICAM-1. Moreover, sh-miR-29b-3p also showed a tendency to enhance the TNFα-activated NF-κB signaling pathway, but there was no statistical difference.

8) The n numbers must be specified for each experiment, especially when SEM is used instead of SD.

We are very sorry for the omission. We have added the number of replicates for the corresponding experiments in the Figure legends of the manuscript.

9) It should be stated that each shown western blot result is representative for at least three independent experiments.

Thank you very much for your professional comment. In the Materials and Methods section of the manuscript, we have highlighted that each shown western blot result is representative for three independent experiments.

Minor
1) The font size in the Figures is sometimes very small. All legends should be clearly readable

We are very sorry for the omission. We have changed the dimensions of some Figures. Since the submission on the system is not a high-resolution image but a manually merged PDF document, we are very sorry that some of the figures in the manuscript may not be clear.

2) Second page of results was the CX3CL1 expression “restored” after sh-CX3CL1 transfection? This should be better explained.

We are very sorry for the mistake. We have replaced “restored” with “inhibited” in the manuscript.

3) Page numbers are missing and would have made the review easier.

We are very sorry for the omission. In the resubmitted revised manuscript, we have added page numbers.

#Reviewer 2 Advance Summary and Potential Significance to Field:
In the study by Li and co-workers, the authors examined a mechanism by which laminar shear stress (LSS) modulates adhesion molecule expression and monocyte adhesion in endothelial cells. Specifically, they examined whether miR-29b-3p, an miRNA that has increased expression in endothelial cells subjected to LSS, suppresses expression of VCAM-1 and ICAM-1 through targeting of CX3CL1. The authors show that expression of CX3CL1, VCAM-1, and ICAM-1 was decreased in cultured endothelial cells subjected to LSS. Subsequently, they show that short hairpin RNA against CX3CL1 suppressed TNFalpha-induced expression of VCAM-1 and ICAM-1 and monocyte adhesion to endothelial cells. In silico analysis revealed that miR-29b- 3p could potentially target CX3CL1 3’UTR, which was verified in luciferase reporter assays of wild type and mutant CX3CL1 3’UTRs. Enhanced expression of miR-29b in cultured endothelial cells decreased TNFalpha-induced expression of CX3CL1 and downstream mediators of inflammation VCAM-1 and ICAM-1. The authors provide evidence that the mechanism by which LSS and miR-29b suppressed monocyte adhesion to cultured endothelial cells involved inhibition of NF-kappaB signaling. Finally, hypercholesterolemic mice that were injected with agomiR-29b via tail vein had reduced aortic expression of CX3CL1, VCAM-1, and ICAM-1 and less aortic atherosclerotic plaques compared to mice injected with negative control agomiR. The study provides solid evidence for a novel role of the miR-29b/CX3CL1 pathway in LSS-mediated changes in endothelial phenotype vitro. However, it would be helpful if the authors could provide more data on the negative control experiments for the in vitro studies. For the in vivo studies, a stronger case for the physiologic relevance of the miR- 29b/CX3CL1 mechanism could be made with data demonstrating that miR- 29b/CX3CL1 expression differed in endothelial cells from aortic segments exposed to LSS compared to those exposed to disturbed flow. The authors should also provide more data showing exogenous miR-29b suppressed CX3CL1 expression in aortic endothelial cells versus whole aortic tissues. Furthermore, the effect of exogenous miR- 29b

© 2022. Published by The Company of Biologists under the terms of the Creative Commons Attribution License (https://creativecommons.org/licenses/by/4.0/).

on lipid levels suggests that the mechanism responsible for the anti-atherosclerotic effect of agomir-miR-29b may not involve the miR-29b/CX3CL1 pathway. Finally, it is not clear how the current study can be reconciled with a study from 2014 published in the International Journal of Cardiology (Zhu et al., vol. 176, pages 764-770), which showed that ApoE-/- mice fed a high fat diet had increased aortic expression of miR- 29b, and miR-29b mimic enhanced ICAM-1 and NFkappaB expression in cultured endothelial cells.

Since we have answered other questions one by one below, we will mainly reply to your last question here. First of all, thank you very much for your professional and valuable comments on our manuscript, which are very helpful to improve the quality of the manuscript. Your question about the inconsistency between our research results and others, our answer is that this is a normal phenomenon. We believe that scientific research is inherently uncertain and diverse, so researchers need to constantly explore and revise. We can only promise you that all experimental data in this study are real and reliable. Thank you again for your contribution to our manuscript.

#Reviewer 2 Comments for the Author:
Specific comments/questions:

1. It is surprising that the authors did not discuss or reference any of the studies of LSS and KLF2; these studies have identified several LSS-responsive miRNAs involved in modulating endothelial cell phenotype.

Thank you very much for your professional comment. We have cited references related to LSS and KLF2 in the revised manuscript (PMID: 29407891, PMID: 25359860 and PMID: 26456066).

2. Based on their in vivo studies, it is not clear that the authors can conclude that LSS inhibits AS progression by regulating CX3CL1. The in vivo experiments do not show association between LSS, miR-29b, and CXCL1 in mouse aortic endothelial cells. In addition, exogenous miR-29b altered lipid levels of the ApoE-/- mice on HFD, so it is not clear that the miR-29b/CX3CL1 pathway is relevant in this model

Thank you very much for your professional comment. Based on your question, we linked Lss to miR-29b-3p/CX3CL1 in vivo using preserved tissue samples. We investigated the expression levels of miR-29b-3p and CX3CL1 in the intima of the aortic arch of ApoE$^{-/-}$ mice exposed to turbulence (Oss) and the intima of the descending thoracic aorta exposed to Lss. As shown in **Figures S3A, B and C**, the expression of miR-29b-3p in the thoracic aortic intima exposed to Lss was up-regulated, whereas the expression of CX3CL1 was down-regulated compared with the aortic arch. This is consistent with in vitro results. In addition, your question of whether exogenous miR-29b regulates AS formation by affecting lipid levels in mice is very enlightening for our next study. However, the changes in lipid levels in mice detected in this study are only used as an evaluation index for AS. As shown in **Figure 6**, exogenous miR- 29b did reduce CX3CL1 expression levels and AS formation in aortic tissue of HFD- fed ApoE$^{-/-}$ mice. Therefore, we believe that CX3CL1 is partly responsible for the regulation of AS development by miR-29b. In follow-up studies, we will continue to explore whether miR-29b-3p affects AS formation through lipid regulation based on your suggestion.

3. Figure 1: it is very difficult to read words in panels A-J, F-G, even after magnification. Please indicate amount LSS to which the cells were exposed and rationale for this amount. In panel E, it is not clear why the number of downregulated genes went from 155 to 130 in HAECs and 853 to 828 in HUVECs.

We are very sorry for the omission. We have changed the dimensions of some Figures. Since the submission on the system is not a high-resolution image but a manually merged PDF document, we are very sorry that some of the figures in the manuscript may not be clear. The Materials and Methods section of the manuscript has demonstrated that the in vitro Lss-treated cell model established in this study can apply a shear stress of 12 dyn/cm^2 to endothelial cells. The Lss of 12 dyn/cm^2 is close to the physiological shear stress of the body and is the shear stress level used by most references in the field of cardiovascular research. In **Figure 1E,** 25 of the down- regulated genes in HAECs and HUVECs were identical (marked in purple). Therefore, the down-regulated genes in HAECs are 155 (130+25), and the down-regulated genes in HUVECs are 853 (828+25).

© 2022. Published by The Company of Biologists under the terms of the Creative Commons Attribution License (https://creativecommons.org/licenses/by/4.0/).

4. Figure 2 shows gene expression and monocyte adhesion in cells with only one negative control. What about the other negative controls for these experiments? For example, data for gene expression in cells treated with TNFalpha plus sh-NC, cells treated with TNFalpha plus LSS and pri-NC should be shown. Please provide western blot data for panel G.

Thank you very much for your professional comment. The negative controls you mentioned all exist in the figures, we are very sorry for not labeling them clearly. For example, in **Figures 2E and F**, the TNFα group represents the TNFα+sh-NC group. Sorry again for our mistakes, we have relabeled the groups in the figures. We have provided the western blot data corresponding to **Figure 2G** in **Figures S3F and G**.

5. Figure 3 – please clarify what was used as negative control mimic for luciferase reporter gene experiment. Please clarify why only the low concentration of sh-miR-29b suppressed miR-29b expression.

Thank you very much for your professional comment. We have added a negative control mimic for the luciferase reporter gene experiment in Results section of the revised manuscript. We also found this interesting phenomenon in our experiments. Not that the higher the plasmid concentration, the lower the expression of miR-29b-3p. We believe that plasmids transfected into cells interact with cells within a certain concentration range. When the concentration of plasmid is high, the interaction range is exceeded, so microRNA expression does not drop as expected.

6. Figure 4 – similar comment about negative control data for pri-miR-NC as stated in #4 above. What about data for TNFalpha-pri-miR-NC or TNFalpha plus LSS and miR- NC? It is not clear that the quantitative data for VCAM-1 in panel G reflect blot in panel F.

We are very sorry for the mistake. The negative controls you mentioned all exist in the figures, we are very sorry for not labeling them clearly. And we have relabeled the groups in the figures. The quantitative histogram of panel G reflected the western blot data of panel F.

7. Figure 5 – it is hard to see from the immunofluorescence images provided in panel F that LSS decreased TNFalpha-induced nuclear translocation of p65. Can clearer or more magnified images be provided? For experiments depicted in panels G-L, it would be helpful to see all of the data for negative control conditions, e.g. TNFalpha plus NC. Also, it would be helpful to see data on TNFalpha plus sh-miR-29b.

1) Since the uploaded figures are not high-resolution original images, the contents of the figures may not be clear. We are very sorry for the inconvenience to your review. We have provided manually enlarged and cropped images below.

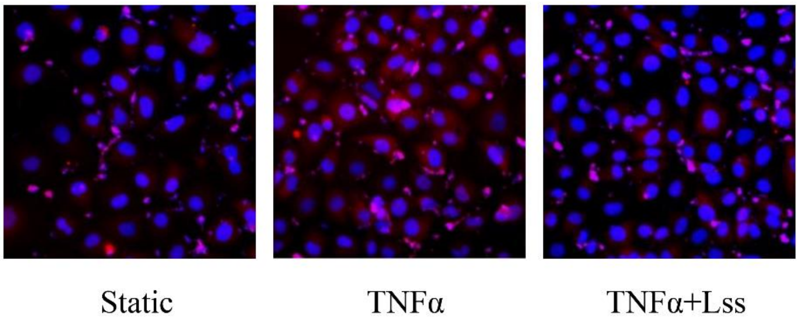

2) Based on your comments, we have supplemented the corresponding negative controls in **Figure S4**.

8. Figure 6 – please clarify whether the sequences for mouse and human miR-29b are homologous and whether mouse and human CX3CL1 are homologous. Please provide data for mice injected with negative control agomiR. Since in vitro studies showed that miR-29b suppressed monocyte adhesion to endothelial cells, can the authors provide information on monocyte infiltration into vessel wall in agomiR-treated mice? Please clarify why lipid levels are altered by exogenous miR-29b and how

© 2022. Published by The Company of Biologists under the terms of the Creative Commons Attribution License (https://creativecommons.org/licenses/by/4.0/).

this might impact findings. Please provide additional information on how plaque area and lipid deposition area was quantified in whole aortas of mice. Since the analysis of gene expression involved whole aorta tissues, it is not clear whether miR-29b reduced endothelial cell inflammation in vivo.

1) Thank you very much for your professional comment. We found that hsa-miR-29b- 3p and mmu-miR-29b-3p were homologous by querying the miRBase database. Similarly, based on the NCBI HomoloGene database, we found that mouse CX3CL1 and human CX3CL1 are homologous.
2) The ND group and the HFD group in **Figure 6** were the corresponding data of mice injected with the negative control agomir-NC (ND+ agomir-NC, HFD+ agomir- NC). We are very sorry for not labeling the pictures correctly. And we have relabeled in the revised manuscript.
3) According to the current experimental technical capabilities of our research group, we are very sorry that we cannot provide relevant data that can visually display the adhesion and infiltration of monocytes at the animal level.
4) Since the blood lipid level is a key index for clinical evaluation of AS, the lipid level change involved in this study is only an index used to evaluate the formation of AS in mice. Your comments are very enlightening for our follow-up research, and we may further study the molecular mechanism of miR-29b-3p affecting lipid metabolism based on your suggestion.
5) Since the HE staining in **Figure 6** is cross-section staining, the plaque distribution of the whole aorta cannot be evaluated, but only the plaque distribution of the current cross-section. However, multiple cross-sections were randomly selected from each mouse's aortic tissue, so the results presented in **Figure 6** are representative of most plaque formation in the aorta. The lipid deposition in the aorta can refer to the gross Oil red O stained specimen in **Figure 6I**.
6) Both RNAs and proteins from aortic tissue used in this study were extracted from the aortic intima. And the main components of the aortic intima are endothelial cells. As shown in **Figures 6J and K**, exogenous miR-29b-3p can alleviate HFD-induced endothelial cell inflammation. In addition, the immunohistochemical results in **Figure 6L** also showed that exogenous miR-29b-3p could reduce the protein expression level of VCAM-1 in the aortic intima.

9. In the Discussion section, first paragraph, the authors state that the pharmacologic mechanism of statins in treatment of AS is to improve the effect of turbulence on endothelial cell function by inhibiting activity of YAP/TAZ. This statement is not accurate since it is well recognized that the primary pharmacologic mechanism of statins in treatment of AS is lipid lowering.

Thank you very much for your professional comment. Based on your question, we have removed the relevant references and corresponding content in the revised manuscript.

Second decision letter

MS ID#: JOCES/2021/259696

MS TITLE: Laminar shear stress alleviates monocyte adhesion and atherosclerosis development via miR-29b-3p/CX3CL1 axis regulation

AUTHORS: Luya Pu, Qingyu Meng, Shuai Li, Yaru Wang, Banghao Sun, Bin Liu, and Fan Li
ARTICLE TYPE: Research Article

We have now reached a decision on the above manuscript.

To see the reviewers' reports and a copy of this decision letter, please go to: https://submit-jcs.biologists.org and click on the 'Manuscripts with Decisions' queue in the Author Area. (Corresponding author only has access to reviews.)

As you will see, the reviewers gave favourable reports but raised some points that will require amendments to your manuscript. In particular it is important to address the concerns of reviewer 2. I hope that you will be able to carry these out because I would like to be able to accept your paper, depending on further comments from reviewers.

© 2022. Published by The Company of Biologists under the terms of the Creative Commons Attribution License (https://creativecommons.org/licenses/by/4.0/).

Please ensure that you clearly highlight all changes made in the revised manuscript. Please avoid using 'Tracked changes' in Word files as these are lost in PDF conversion.

I should be grateful if you would also provide a point-by-point response detailing how you have dealt with the points raised by the reviewers in the 'Response to Reviewers' box. Please attend to all of the reviewers' comments. If you do not agree with any of their criticisms or suggestions please explain clearly why this is so.

Reviewer 1

Advance summary and potential significance to field

Thank you for the detailed reply.
An important technical concern remains.

Comments for the author

The first original work from Bazan et al. in Nature and the vast majority of subsequent papers on CX3CL1 identified the full length molecule at 95 KDa. It may be that the detection of a smaller protein in the present manuscript and other studies is a result of a particular antibody. This needs to be carefully checked and described in the manuscript. Discrepancies to the literature need to be discussed carefully. The CX3CL1 protein consists of approximately 400 aa. If at all the 42 kDa could represent unglycosylated CX3CL1. However, the mucin stalk was consistently reported to be heavily O-glycosylated and not unglycosylated. In the full blot for Fig 1 provided a prominent 35 kDa protein was detected as well and it is not clear whether this is another CX3CL1 variant. Moreover, there is no difference visible for the 42 and 35 kDa proteins between the two lanes as it was suggested in Fig 1. The correct full blot should be provided as a supplemental figure in the manuscript, referenced and explained in the results section.

Reviewer 2

Advance summary and potential significance to field

In their revised manuscript, Li and co-authors have been responsive to reviewer concerns regarding in vitro experiments. They convincingly show that LSS-sensitive miR-29b-3p could regulate expression of CX3CL1 in cultured human aortic endothelial cells subjected to TNFalpha. Their data also show that suppression of CX3CL1 by miR-29b-3p inhibited NFkappaB signaling, decreased VCAM-1 and ICAM-1 expression, and decreased binding of monocytes to endothelial cells. However, some concerns about the in vivo model remain. In their response, the authors state that intima of mouse aorta was studied for gene expression, but it is not clear whether this response refers only to the experiment depicted in Figure SA, B, and C or also for gene expression data depicted in Figure 6. If intima was not studied in the experiment depicted in Figure 6 then it should be, and, ideally, gene expression in non-intimal part of vessel wall should be studied for comparison. If intima was studied in Figure 6, then the authors should provide a description of how intima was isolated in the Methods section. There are still concerns about the proposed mechanism for suppression of vascular inflammation and atherosclerosis by exogenous miR-29b in vivo. In their model, it is likely that there was significant uptake of exogenous miR-29b by the liver. Based on the data and modest changes in CX3CL1, it is quite possible that exogenous miR-29b suppressed vascular inflammation and atherosclerosis through suppression of lipid metabolism in liver cells. This possibility should be addressed by the authors in the manuscript.

Comments for the author

Specific Comments/Questions:
1. Please clarify whether turbulent flow (oscillatory shear stress) increased CXCL1 and decreased miR-29b in vitro?
2. Please clarify what happens to NFkappaB signaling in cells treated with TNFalpha, LSS, and sh-miR-29. In Figure S2D, it is not clear whether miR-29b can regulate IkappaBalpha.

© 2022. Published by The Company of Biologists under the terms of the Creative Commons Attribution License (https://creativecommons.org/licenses/by/4.0/).

3. Please clarify the method for aortic intima isolation and which experiments depict gene expression in aortic intima. For example, the manuscript and figure legends refer to gene expression in “aortic tissues,” which suggest whole aortic tissues. Is there expression of CX3CL1 and miR-29b in non-intimal (muscular) part of the aortic tissues?
4. Was exogenous miR-29b taken up by liver after tail vein injections? Could miR-29b be suppressing expression of genes in liver cells?

Minor:
1. In abstract, the authors state, “inflammatory infiltration and plaque formation in aorta, which were significantly reduced after agomir-miRNA-29b-3p injection in ApoE-/- mice fed a HFD via the tail vein.” This sentence sounds mice were fed food through their tail vein.

Second revision

Author response to reviewers' comments

#Reviewer 1 Comments for the Author:
The first original work from Bazan et al. in Nature and the vast majority of subsequent papers on CX3CL1 identified the full length molecule at 95 KDa. It may be that the detection of a smaller protein in the present manuscript and other studies is a result of a particular antibody. This needs to be carefully checked and described in the manuscript. Discrepancies to the literature need to be discussed carefully. The CX3CL1 protein consists of approximately 400 aa. If at all the 42 kDa could represent unglycosylated CX3CL1. However, the mucin stalk was consistently reported to be heavily O-glycosylated and not unglycosylated. In the full blot for Fig 1 provided a prominent 35 kDa protein was detected as well and it is not clear whether this is another CX3CL1 variant. Moreover, there is no difference visible for the 42 and 35 kDa proteins between the two lanes as it was suggested in Fig 1. The correct full blot should be provided as a supplemental figure in the manuscript, referenced and explained in the results section.

Thank you very much for your professional comment. And we are very sorry for the wrong explanation given in the last reply. We have discussed differences in the molecular weight of CX3CL1 from other literature, and have cited and interpreted the full western blot of CX3CL1 **(Figure S2I)** in the revised manuscript. In addition, we have replaced the Western blot of **Figure 1P** and the corresponding original uncropped blot. Due to the low specificity of the antibody, a significant 35 kDa protein was also detected in the full blot.

It should be noted that since the initial experimental design of this study was to explore the differences in gene and protein expression between “Static group”, “Lss group” and “24h recovery after Lss group”, the original uncropped western blot in **Figure 1** has three sets of data. However, in the follow-up study, it was found that the statistical difference of “24h recovery after Lss group” was not significant, so only “Static group” and “Lss group” were kept.

Reviewer 2 Advance Summary and Potential Significance to Field:
In their revised manuscript, Li and co-authors have been responsive to reviewer concerns regarding in vitro experiments. They convincingly show that LSS-sensitive miR-29b-3p could regulate expression of CX3CL1 in cultured human aortic endothelial cells subjected to TNFalpha. Their data also show that suppression of CX3CL1 by miR- 29b-3p inhibited NFkappaB signaling, decreased VCAM-1 and ICAM-1 expression, and decreased binding of monocytes to endothelial cells. However, some concerns about the in vivo model remain. In their response, the authors state that intima of mouse aorta was studied for gene expression, but it is not clear whether this response refers only to the experiment depicted in Figure SA, B, and C or also for gene expression data depicted in Figure 6. If intima was not studied in the experiment depicted in Figure 6, then it should be, and, ideally, gene expression in non-intimal part of vessel wall should be studied for comparison. If intima was studied in Figure 6, then the authors should provide a description of how intima was isolated in the Methods section. There are still concerns about the proposed mechanism for suppression of vascular inflammation and atherosclerosis by exogenous miR-29b in vivo. In their model, it is likely that there was significant uptake of exogenous miR-29b by the liver. Based on the data and modest changes in CX3CL1, it is quite possible that exogenous miR-29b suppressed vascular inflammation and

© 2022. Published by The Company of Biologists under the terms of the Creative Commons Attribution License (https://creativecommons.org/licenses/by/4.0/).

atherosclerosis through suppression of lipid metabolism in liver cells. This possibility should be addressed by the authors in the manuscript.

#Reviewer 2 Comments for the Author:
Specific Comments/Questions:

1) Please clarify whether turbulent flow (oscillatory shear stress) increased CXCL1 and decreased miR-29b in vitro?

Thank you very much for your professional comment. According to your suggestion, the equipment in **Figure S5A** was used to simulate the in-vitro turbulence environment (The equipment was purchased from Shanghai Naturethink Life & Scientific Co., Ltd.). It has been reported in the literatures that oscillating shear stress can be simulated by applying a shear stress of 0.5 ± 4 dyn/cm2 to endothelial cells in the in-vitro simulation equipment (PMID: 32169277 and PMID: 33468662). In vitro studies found that the expression of CX3CL1 was up-regulated in HAECs treated with oscillatory shear stress, while the expression of miR-29b-3p was down-regulated (**Figures S5B and C**).

2) Please clarify what happens to NFkappaB signaling in cells treated with TNFalpha, LSS, and sh-miR-29. In Figure S2D, it is not clear whether miR-29b can regulate IkappaBalpha.

Thank you very much for your professional comment. As shown in **Figures S5D and E**, the phosphorylation of p65 and IκBα induced by TNF-α were inhibited after loading Lss, while knockdown of miR-29b-3p reversed this inhibition. In addition, we have replaced the western blot of p-IκBα in **Figure S2D**.

3) Please clarify the method for aortic intima isolation and which experiments depict gene expression in aortic intima. For example, the manuscript and figure legends refer to gene expression in “aortic tissues,” which suggest whole aortic tissues. Is there expression of CX3CL1 and miR-29b in non-intimal (muscular) part of the aortic tissues?

Thank you very much for your professional comment. We have added the aortic intima isolation method to the Materials and Methods section of the revised manuscript. Both qRT-PCR assay and Western blotting assay in the in-vivo experimental part were used to analyze the gene and protein expression levels of the aortic intima. And we have revised the inappropriate description in the manuscript and figure legends. It has been reported that CX3CL1 (PMID: 12824004, PMID: 29356931 and PMID: 24788416) and miR-29b-3p (PMID: 33761588) are expressed in vascular smooth muscle cells. Since this study was aimed at the regulation mechanism of aortic endothelial cells and aortic intima, other tissue samples of the aorta were not preserved. We are very sorry for not being able to provide the corresponding experimental data. Your question has inspired us a lot, and we will explore gene expression differences in the non-intima part of aortic tissue in the follow-up study.

4) Was exogenous miR-29b taken up by liver after tail vein injections? Could miR-29b be suppressing expression of genes in liver cells?

Thank you very much for your professional comment. It has been reported that miR- 29b-3p was expressed in the liver (PMID: 29091295 and PMID: 29403377). However, since mouse liver tissues were not preserved and the in-vivo experiment period was too long, we are very sorry that we cannot provide the corresponding experimental data. We have discussed the possibility of miR-29b-3p regulating liver lipid metabolism in the Discussion section of the manuscript. Your comments have inspired us a lot, and in the follow-up study we will delve into the effects of miR-29b-3p on the liver based on your suggestions.

Minor:

1) In abstract, the authors state, “inflammatory infiltration and plaque formation in aorta, which were significantly reduced after agomir-miRNA-29b-3p injection in ApoE−/− mice fed a HFD via the tail vein.” This sentence sounds mice were fed food through their tail vein.

© 2022. Published by The Company of Biologists under the terms of the Creative Commons Attribution License (https://creativecommons.org/licenses/by/4.0/).

We are very sorry for the incorrect description. We have replaced “inflammatory infiltration and plaque formation in aorta, which were significantly reduced after agomir-miRNA-29b-3p injection in ApoE$^{-/-}$ mice fed a HFD via the tail vein.” with “inflammatory infiltration and plaque formation in aorta, which were significantly reduced after injection of agomir-miRNA-29b-3p via the tail vein into HFD-fed ApoE$^{-/-}$ mice.” in the revised manuscript.

Third decision letter

MS ID#: JOCES/2021/259696

MS TITLE: Laminar shear stress alleviates monocyte adhesion and atherosclerosis development via miR-29b-3p/CX3CL1 axis regulation

AUTHORS: Luya Pu, Qingyu Meng, Shuai Li, Yaru Wang, Banghao Sun, Bin Liu, and Fan Li
ARTICLE TYPE: Research Article

I am happy to tell you that your manuscript has been accepted for publication in Journal of Cell Science, pending standard ethics checks.

© 2022. Published by The Company of Biologists under the terms of the Creative Commons Attribution License (https://creativecommons.org/licenses/by/4.0/).
